# *HvLUX1* is a candidate gene underlying the *early maturity 10* locus in barley: phylogeny, diversity, and interactions with the circadian clock and photoperiodic pathways

**DOI:** 10.1111/nph.12346

**Published:** 2013-06-03

**Authors:** Chiara Campoli, Artem Pankin, Benedikt Drosse, Cristina M Casao, Seth J Davis, Maria von Korff

**Affiliations:** 1 Max Planck Institute for Plant Breeding ResearchCarl von Linné Weg 10, D50829, Cologne, Germany; 2 Department of Biology, University of YorkYO10 5DD, York, UK

**Keywords:** barley (*Hordeum vulgare*), circadian clock, *early maturity 10*, gene duplication, *LUX ARRHYTHMO*, photoperiodic flowering, *Ppd-H1*

## Abstract

- Photoperiodic flowering is a major factor determining crop performance and is controlled by interactions between environmental signals and the circadian clock. We proposed *Hvlux1*, an ortholog of the Arabidopsis circadian gene *LUX ARRHYTHMO*, as a candidate underlying the *early maturity 10* (*eam10*) locus in barley (*Hordeum vulgare* L.).
- The link between *eam10* and *Hvlux1* was discovered using high-throughput sequencing of enriched libraries and segregation analysis. We conducted functional, phylogenetic, and diversity studies of *eam10* and *HvLUX1* to understand the genetic control of photoperiod response in barley and to characterize the evolution of *LUX*-like genes within barley and across monocots and eudicots.
- We demonstrate that *eam10* causes circadian defects and interacts with the photoperiod response gene *Ppd-H1* to accelerate flowering under long and short days. The results of phylogenetic and diversity analyses indicate that *HvLUX1* was under purifying selection, duplicated at the base of the grass clade, and diverged independently of *LUX*-like genes in other plant lineages.
- Taken together, these findings contribute to improved understanding of the barley circadian clock, its interaction with the photoperiod pathway, and evolution of circadian systems in barley and across monocots and eudicots.

## Introduction

Barley (*Hordeum vulgare* L.) is an important crop cultivated in a wide range of environments and has recently emerged as a model species for Triticeae as a result of its diploid nature and abundance of genomic resources (International Barley Sequencing Consortium (IBSC), 2012). Genetic variation in photoperiod response was crucial for the successful expansion of barley cultivation from its origin in the Fertile Crescent to northern latitudes (Turner *et al*., 2005; Jones *et al*., 2008). Barley is a facultative long-day plant; long days (LDs) promote flowering in spring, while short days (SDs) delay reproductive development. Flowering under LDs is controlled by the major photoperiod response *PHOTOPERIOD 1* gene (*Ppd-H1*) (Turner *et al*., 2005). *Ppd-H1* is homologous to *PSEUDO-RESPONSE REGULATOR* (*PRR*) genes implicated in the circadian clock of the model species *Arabidopsis thaliana* (hereafter Arabidopsis, Turner *et al*., 2005). The dominant allele of *Ppd-H1* is prevalent in the wild barley progenitor *H. vulgare* spp. *spontaneum* and in Mediterranean cultivated barley genotypes and accelerates flowering under LDs, as an adaptation to short growing seasons. A natural mutation in the conserved CCT domain of *Ppd-H1* causes a reduced response to LDs and was selected for adaptation to long growing seasons (Turner *et al*., 2005; von Korff *et al*., 2006, 2010; Jones *et al*., 2008; Wang *et al*., 2010). Under LD conditions, *Ppd-H1* up-regulates *HvFT1*, which is the barley counterpart of the Arabidopsis ‘florigen’ *FLOWERING LOCUS T* (*FT*). Delayed flowering in genotypes with a mutated *ppd-H1* allele was associated with reduced expression of *HvFT1* (Turner *et al*., 2005; Campoli *et al*., 2012a).

Barley *early maturity* mutants (*eam*) with a reduced or no response to photoperiod have been isolated and used in Swedish, Australian, and South American breeding programs to adapt cultivars to short growing seasons (Laurie *et al*., 1995; Lundqvist, 2009; Zakhrabekova *et al*., 2012). These *eam* mutants have been introgressed into the spring barley cv Bowman (Druka *et al*., 2011) and represent a valuable resource to decipher the genetic control of photoperiod response in the model crop barley. Recently, *HvELF3*, a homolog of the Arabidopsis circadian clock regulator *EARLY FLOWERING 3* (*ELF3*), was identified as a gene underlying the *eam8* quantitative trait locus (QTL), which causes a day-neutral early flowering phenotype (Faure *et al*., 2012; Zakhrabekova *et al*., 2012). Interestingly, the mutation in *HvELF3* caused an up-regulation of *Ppd-H1* and the downstream *HvFT1* under noninductive SD conditions (Faure *et al*., 2012). In addition, the *Hvelf3* mutants were severely compromised in the expression of clock oscillator and output genes. This study suggested that circadian clock homologs play an important role in the control of flowering in barley. Circadian clocks synchronize biological processes with the diurnal cycle, using molecular mechanisms that include interlocked transcriptional feedback loops. In Arabidopsis, the circadian clock is composed of three negative feedback loops: the inhibition of evening complex (EC) genes *ELF3*, *EARLY FLOWERING 4* (*ELF4*), and *LUX ARRHYTHMO* (*LUX*, also known as *PHYTOCLOCK1*) by the rise of CIRCADIAN CLOCK ASSOCIATED1 (CCA1) and LATE ELONGATED HYPOCOTYL (LHY) late at night; the inhibition of *PRR* genes by the EC early at night; and the inhibition of *LHY*/*CCA1* by TIMING OF CAB EXPRESSION1 (TOC1) in the morning (Kolmos *et al*., 2009; Huang *et al*., 2012; Nagel & Kay, 2012; Pokhilko *et al*., 2012). Furthermore, the evening-expressed GIGANTEA (GI) protein was modeled as a negative regulator of the EC, which in turn inhibits *TOC1* expression (Herrero *et al*., 2012; Pokhilko *et al*., 2012).

Campoli *et al*. (2012b) have shown that circadian clock genes are structurally conserved between barley and Arabidopsis, and their circadian expression patterns suggested conserved functions. However, phylogenetic analyses revealed that duplications/deletions of clock genes occurred throughout the evolution of eudicots and monocots. For example, the ancient three *PRR* clades expanded independently in monocots and eudicots, supposedly as a result of paleoduplications (Takata *et al*., 2010; Campoli *et al*., 2012b). In monocots, these events gave rise to two paralogous pairs of *PRR* genes, termed *PRR37*/*73* and *PRR59*/*95*. In this context, it is noteworthy that *PRR37* orthologs in monocots, *PPD1* in barley and wheat (Turner *et al*., 2005; Beales *et al*., 2007) and *SbPRR37* in sorghum (*Sorghum bicolor*) (Murphy *et al*., 2011), are major determinants of photoperiod sensitivity and flowering time, while natural variation in *PRR* genes in Arabidopsis did not have a strong effect on flowering time (Ehrenreich *et al*., 2009). These results indicated that the molecular function of genes participating in the circadian clock and photoperiod response diverged and specialized in a lineage-specific manner. Because of the prominent role of photoperiod insensitivity in breeding, it is essential to identify the genetic components controlling this pathway in temperate cereals.

We present the characterization of the barley *eam10* mutation, which causes an early-flowering phenotype under both SDs and LDs. *eam10* was described as an X-ray-induced mutation in Super Precoz (2H) of unknown parentage (Favret & Ryan, 1966; Gallagher *et al*., 1991; Gallagher & Franckowiak, 1997) and was mapped onto chromosome 3HL (Börner *et al*., 2002). Using a high-throughput sequencing approach, we identified the candidate gene underlying the *eam10* locus in barley as *HvLUX1*, a barley ortholog of the Arabidopsis circadian clock regulator *LUX*. We demonstrated that *eam10* caused circadian defects and interacted with the major barley photoperiod response gene *Ppd-H1* to accelerate flowering under LD and SD conditions. Based on the results of phylogenetic and diversity analyses, we conclude that *HvLUX1* was under purifying selection, duplicated at the base of the grass clade, and diverged independently of the *LUX*-like genes in other plant lineages.

## Materials and Methods

### Plant material and growth conditions

Flowering time (days to emergence of the main spike awns) of the barley spring cv Bowman and three Bowman-derived introgression lines Bowman(*Ppd-H1*), Bowman(*eam10*), and Bowman(*Ppd-H1 *+ *eam10*) (kindly provided by R. Waugh, James Hutton Institute, and by David Laurie, John Innes Centre, UK) was recorded for 15–18 plants per genotype. To score flowering, plants were grown in soil in a glasshouse under SDs (8 h light, 20°C : 16 h dark, 18°C) and LDs (16 h light, 20°C : 8 h dark, 18°C). To investigate expression levels of *HvFT1*, plants were sampled 2 h before light-off after 14 and 28 d under LDs and SDs, respectively. Diurnal and circadian expression of core clock and flowering time genes was tested under SD and free-running conditions in Bowman and Bowman(*eam10*). Plants were grown in soil in a controlled-environment growth chamber (CEGC). After 21 d under SDs, leaf material was harvested every 2 h for a total of 24 h from the start of the light period (time point T0). Night samples (T10 to T22) were collected in the dark. After SDs, plants were released into continuous light and constant temperature (20°C; LL) and sampled every 2 h for 48 h, starting after 8 h of continuous light (T8). Two biological replicates, comprising the second youngest leaves of three independent plants, were analyzed. All samples were immediately frozen in liquid nitrogen and stored at −80°C until processed.

Meristem development was scored in a separate experiment with Bowman and Bowman(*eam10*) grown in soil in a CEGC. The main stem of three plants per genotype was dissected starting 7 d after germination every 2–3 d under LDs and every 3–4 d under SDs until flowering. After day 79 under SDs the experiment was stopped when the shoot apical meristems of the remaining plants had died. Meristem development was scored following the Waddington scale (Waddington *et al*., 1983). To generate developmental series for the analysis of *HvFT1* expression in the leaf and to link it to specific meristem stages, two biological replicates were harvested 2 h before light-off at every developmental stage under both photoperiods.

To investigate natural diversity of a candidate gene, a set of 88 wild (*H. vulgare* ssp. *spontaneum* and *H. agriocrithon*) and cultivated barley genotypes was selected from germplasm collections of the Max Planck Institute of Plant Breeding Research in Cologne, Germany (Badr *et al*., 2000) and the Barley 1K collection (Hübner *et al*., 2009; Supporting Information, Table S2).

### Gene expression analysis

Gene expression was analyzed in leaf samples harvested from Bowman, Bowman(*Ppd-H1*), Bowman(*eam10*), and Bowman(*Ppd-H1 *+ *eam10*) grown under LDs and SDs, from a developmental series of leaf samples from Bowman and Bowman(*eam10*), and from diurnal and circadian sampling of Bowman and Bowman(*eam10*) leaves. Total RNA extraction, cDNA synthesis, and quantitative reverse transcription polymerase chain reactions (qRT-PCRs) using gene-specific primers were performed as explained in Campoli *et al*. (2012b). Expression of *HvLUX1* was analyzed using the following primer combinations: LUX_1077F 5′-AATTCAGTCCACGGATGCTC-3′ and LUX_1289R 5′-CTTCACTTCAGCTCCCCTTG-3′.

### Identification and characterization of a candidate gene underlying *eam10*

To identify a candidate gene for the *eam10* QTL, we sequenced a set of flowering-related genes from the genotypes Bowman and Bowman(*eam10*) using high-throughput sequencing of multiplexed enriched libraries. A sample of pooled barcoded TruSeq libraries was prepared following the standard Illumina protocol with modifications (Methods S1). The pooled library was enriched for flowering-related genes using the SureSelect target enrichment system (Agilent Technologies, Böblingen, Germany) according to the manufacturer’s recommendations with the following modification. To alleviate biased PCR amplification of GC-rich regions, the post-enrichment amplification of the library was performed using the ‘long denaturation’ Phusion (Thermo Scientific, St Leon-Rot, Germany) protocol (Aird *et al*., 2011). The enriched library was paired-end-sequenced on a HiSeq 2000 machine (Illumina, San Diego, CA, USA).

The genes for targeted enrichment were selected by following two approaches. First, known flowering-related barley genes and gene families were directly extracted from NCBI GenBank (Table S3a). Second, additional barley genes were selected based on their homology with the genes of interest from *Brachypodium distachyon* as determined by the Blastx search in HarvEST assembly 35 (http://harvest.ucr.edu) (Table S3b). The nomenclature and annotation of Brachypodium genes were as reported by Higgins *et al*. (2010). Selected barley genes were used as a template to design a library of baits for the solution-based target enrichment approach and as a reference target genome for mapping in subsequent bioinformatics analyses.

Read datasets were preprocessed using a set of filters (adapter trimming, quality and length, sequencing artefacts, contamination) using the Galaxy server at Wageningen University, the Netherlands (http://galaxy.wur.nl; Methods S2) and mapped to the reference target genome as a single-end dataset using BWA 0.5.9 with customized parameters: ‘-n’ 0.03, ‘-o’ 2, ‘-e’ 10 (Li & Durbin, 2009a). Reads mapped to several locations and PCR duplicates were removed using ‘view’ and ‘rmdup’ functions of SAMtools 0.1.18 (Li *et al*., 2009b). Mean depth of coverage was estimated using BAMstats 1.25 (http://bamstats.sourceforge.net). Polymorphisms were discovered using ‘IndelRealigner’ and ‘UnifiedGenotyper’ algorithms of the GATK 2.1.3 package (DePristo *et al*., 2011). A set of stringent ‘hard’ filters was applied to the raw single nucleotide polymorphism (SNP) set to select reliable SNPs (Methods S3).

Genes polymorphic between Bowman and Bowman(*eam10*) were located on barley linkage groups using GenomeZipper (Mayer *et al*., 2011). The location of the *eam10* QTL on the barley consensus genetic map (OPA123-2008 consensus; Close *et al*., 2009) was extracted from the GrainGenesCMap browser (http://wheat.pw.usda.gov/cgi-bin/cmap). The position of the MYB domain on HvLUX1 was determined using InterProScan (Quevillon *et al*., 2005).

### Segregation analysis

A set of 1002 BC_1_F_2_ plants derived from backcrossing Bowman(*eam10*) to Bowman were grown in the field in Cologne in 2012 (spring sowing). A total of 215 (21%) plants were scored as early flowering and seeds were harvested. Early-flowering BC_1_F_2:3_ lines were sown in the glasshouse under LDs. Leaf samples were harvested and flowering time was scored. Barley genomic DNA was extracted using the BioSprint 96 kit (Qiagen, Hilden, Germany) according to the manufacturer’s recommendations and quantified using Quant-iT™ PicoGreen assay (Invitrogen) measured using the Synergy™ 4 microplate reader (Biotek, Bad Friedrichshall, Germany). A full-length coding sequence (CDS) of *HvLUX1* was amplified using specific primers LUX_135F_T3 5′-aattaaccctcactaaagggTGGCGAGGGTAAGTTGATTC-3′ LUX_1108R_T7 5′-gtaatacgactcactatagggcGAGCAGAGAGCAGAGCATCC-3′ with T3 and T7 overhangs for sequencing (in lower case). PCR reactions (0.5× HF buffer, 0.16 μM dNTPs, 0.5 μM primers, 0.8 U Phusion Hi-Fi polymerase (Thermo Scientific), 100 ng DNA) were incubated in the PTC DNA Engine thermocycler (Bio-Rad, Hercules, CA, USA) under the following conditions: 95°C for 3 min; 30 cycles of 95°C for 20 s, 60°C for 30 s, 72°C for 1 min; 72°C for 5 min. PCR fragments were purified using the QIAquick PCR purification kit following the manufacturer’s recommendations and Sanger-sequenced. The sequences were assembled using SeqMan software (DNASTAR Lasergene® 8 Core Suite, Madison, WI, USA).

### Multiple alignment and phylogeny reconstruction

A barley cDNA, *HvLUX2* homologous to *HvLUX1* was extracted from NCBI GenBank using Blastn search in the nr/nt database and, because of the misannotation of the *LUX2* cDNA in GenBank, it was manually translated. HvLUX1 and HvLUX2 proteins were further used as NCBI Blastp queries to extract homologs from other plant species (E-value cutoff 10^−30^; last search on 22 November 2012). In addition, LUX-like protein from *S. bicolor* (Sb03 g039610) was retrieved from the Gramene database (http://www.gramene.org/Sorghum_bicolor). One representative per taxa was kept in case several identical sequences were retrieved. The nomenclature and accession numbers of analyzed LUX-like sequences (species – genotype (database accession number)) are as follows: *A. thaliana*: At1 – Columbia (LUX1, NP_001190022; NOX1a, NP_200765), At2 – Wassilewskija/Landsberg erecta (NOX1b, AAM65635); *A. lyrata*: Al – MN47 (LUX1, XP_002875796; NOX1, XP_002864640); *B. distachyon*: Bd – Bd21 (LUX1, XP_003565161; LUX2, XP_003567265); *Glycine max*: Gm – Williams 82 (LUX1, XP_003539607; LUX2, XP_003537977); *Lycoris longituba*: Ll – six mixed varieties (LUX1, ADG58105; LUX2, ADG58070; LUX3, ADG57915; LUX4, ADG57989); *Medicago truncatula*: Mt – A17 (LUX1, XP_003606714); *Nicotiana benthamiana*: Nb – unknown (LUX1, BAE16280); *H. vulgare* ssp. *vulgare*: Haruna Nijo (LUX1, BAJ88719; LUX2, AK356714 manually translated); *Hyacinthus orientalis*: Ho – Delft Blue (LUX1, AAS21003); *Oryza sativa* ssp. *indica*: Osi – 93-11 (LUX1, EAY77400; LUX2, EEC71789); *O. sativa* ssp. *japonica*: Osj1 – Zhonghua11 (LUX1a, AAS90600), Osj2 – Nipponbare (LUX1b, NP_001045537; LUX2, NP_001044783); *Physcomitrella patens* ssp. *patens*: Pp – Gransden2004 (LUX1, XP_001757337; LUX2, XP_001768612; LUX3, XP_001757676; LUX4, XP_001757677); *Populus trichocarpa*: Pt – Nisqually-1 (LUX1, XP_002299857; LUX2, XP_002314163); *Ricinus communis*: Rc – Hale (LUX1, XP_002520534); *Selaginella moellendorffii*: Sm – Plants Delights Nursery (LUX1; XP_002962201); *Solanum lycopersicum*: Sl – unknown (LUX1, BAE16281); *S. tuberosum*: St – unknown (LUX1, BAE16282); *S. bicolor*: Sb – BTx623 (LUX1, XP_002459177; LUX2, Sb03 g039610); *Vitis vinifera*: Vv – PN40024 (LUX1, XP_002283159); *Zea mays*: Zm – mixed genotype (LUX1a, NP_001143908; LUX1b, NP_001147359; LUX2, DAA56868). Protein sequences were aligned using MAFFT v.6.851b with the E-INS-i algorithm (Katoh & Toh, 2008). Visually unaligned regions were cropped from the alignment using BioEdit 7.0.9.0 software (Hall, 1999). The alignment was converted from fasta to Phylip4 format using ReadSeq 2.1.30 tool implemented at EBI (http://www.ebi.ac.uk/cgi-bin/readseq.cgi). A best-scoring maximum likelihood tree and pairwise distances were calculated using the fast bootstrapping algorithm of the RAxML 7.3.5 with the GAMMAJTT model and ‘autoMRE’ parameter determining an optimal number of bootstrap replicates (Stamatakis *et al*., 2008). The best-fitting amino-acid substitution model (JTT) was estimated using ProteinModelSelection Perl script (http://www.exelixis-lab.org/software/ProteinModelSelection.pl). The tree was edited in Dendroscope 3 software (Huson & Scornavacca, 2012). Microsynteny analysis at the *LUX* loci in *B. distachyon*, *O. sativa* ssp. *japonica*, and *S. bicolor* was performed using CoGe GEvo script (Lyons & Freeling, 2008) with the default BlastZ parameters except for the score threshold 6000 (Methods S4). The sequence logo of the SHAQKYF motif was generated using WebLogo (Crooks *et al*., 2004).

### Analysis of *HvLUX1* natural diversity

A CDS of *HvLUX1* was amplified and sequenced as indicated in the ‘Segregation analysis’ section (GenBank accession nos. KC668259–KC668274). The sequences were assembled using SeqMan software (DNASTAR Lasergene® 8 Core Suite) and polymorphic haplotypes (sequence variants) were discerned manually based on the revealed polymorphisms. The Median Joining haplotype network was constructed using SplitsTree4 with default parameters (Huson & Bryant, 2006). Indels were encoded as a nucleotide substitution to include them in the analysis as an informative character.

### Statistical analysis

Significant differences in flowering time among Bowman, Bowman(*eam10*), and Bowman(*eam10 *+ *Ppd-H1*) grown under SDs or LDs were calculated using paired *t*-test with a 95% confidence level (*P *< 0.05). Significant differences in meristem development and gene expression between Bowman and Bowman(*eam10*) were calculated using a general linear model in the SAS software 9.1.3 (SAS Institute, 2009) with the factors genotype, time point, biological replicate, and first-order interaction effects. Significant differences (*P* < 0.05) between least-squares means (LSmeans) of the genotype × time interactions were calculated using a Tukey–Kramer adjustment for multiple comparisons.

## Results

### The *eam10* mutant is early flowering under SD and LD conditions

**Figure 1:**
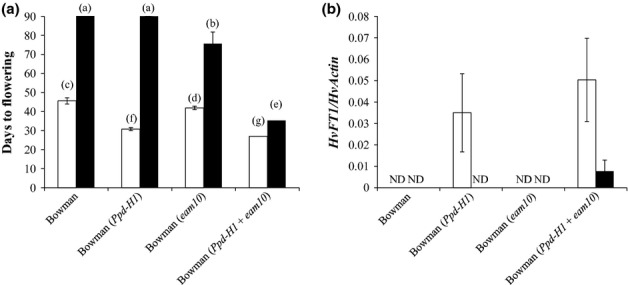
Flowering time and *HvFT1* expression levels of barley (*Hordeum vulgare*) wildtype and introgression lines. Bowman and introgression lines carrying *PHOTOPERIOD 1* gene (*Ppd-H1*), *early maturity 10* (*eam10*) or *Ppd-H1 *+ *eam10* grown under long days (open bars) or short days (closed bars). (a) Flowering time is shown as mean days to emergence of the main spike awns. Error bars represent SD for 15–18 plants of each genotype. Genotypes with the same letter are not significantly different by paired *t*-test. (b) *HvFT1* expression relative to *HvActin* is shown as average of two biological and two technical replicates ± SD. Leaf material was sampled after 2 wk (long days) and 4 wk (short days) 2 h before light-off. ND, not detectable.

In this study, we analyzed the effect of *eam10* on photoperiod-dependent flowering in barley and its interaction with natural variation in the major barley photoperiod response gene *Ppd-H1*. Flowering time was scored in the spring cv Bowman(*ppd-H1*) and in three derived introgression lines Bowman(*eam10*), Bowman(*Ppd-H1*), and Bowman(*Ppd-H1 *+ *eam10*) under LDs and SDs (Fig. 1a). Under LDs, Bowman(*Ppd-H1 *+ *eam10*) flowered first at 27 d after sowing (DAS), followed by Bowman(*Ppd-H1*) at 31 DAS, Bowman(*eam10*) at 42 DAS, and Bowman at 46 DAS. Under SDs, Bowman and Bowman(*Ppd-H1*) did not flower until 90 DAS when the experiment was terminated. By contrast, Bowman(*eam10*) and Bowman(*Ppd-H1*+*eam10*) flowered under SDs at 76 and 35 DAS, respectively. Thus, *eam10* accelerated flowering under both LDs and SDs. The combination of *Ppd-H1* and *eam10* resulted in the earliest flowering phenotype irrespective of the photoperiod. Interestingly, variation at *Ppd-H1* caused a significant difference in flowering time in the background of *eam10* under SDs. Taken together, these findings suggest that *Ppd-H1* interacts epistatically with *eam10* to accelerate flowering under both LD and SD conditions.

**Figure 2:**
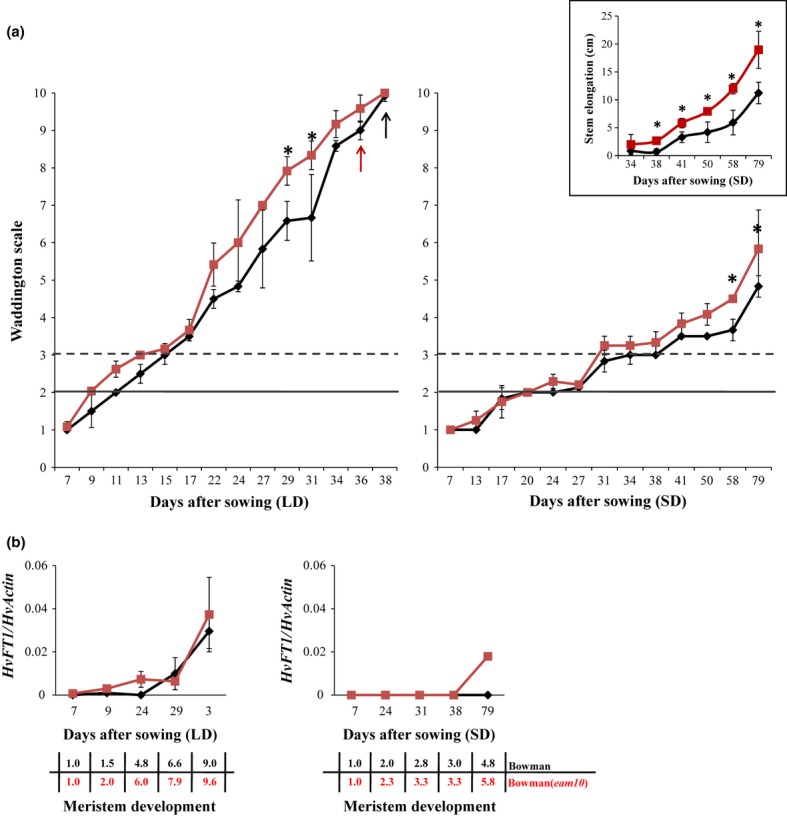
Meristem development and expression levels of *HvFT1* under long days and short days in barley (*Hordeum vulgare*) Bowman and Bowman(*eam10*) plants. (a) Apex development assessed by the Waddington scale under long- and short-day conditions in Bowman (black lines) and Bowman(*eam10*) (red lines). Timings of double ridge formation (WS2) and the initiation of internode elongation (WS3) are indicated by horizontal full and dashed lines, respectively. Black and red arrows indicate the day when Bowman and Bowman(*eam10*) headed under long-day conditions. The small chart above shows the distance in cm between the base of the stem and the base of the meristem measured under short-day conditions on the indicated days. Values are means of three plants ± SD. Significant differences in meristem development and stem elongation are indicated: *, *P* < 0.05. (b) *HvFT1* expression relative to *HvActin* in Bowman (black lines) and Bowman(*eam10)* plants (red lines) under long- and short-day conditions. The second last leaves of three plants were harvested and pooled. Sampling was done 2 h before light-off. The average Waddington stage of meristems at the respective sampling times is reported in the table. Values represent the average of two biological and two technical replicates ± SD.

To determine the effect of *eam10* on the development of the shoot apical meristem (SAM), we dissected meristems of Bowman and Bowman(*eam10*) plants starting 1 wk after germination until heading and scored morphological changes of the SAM based on the Waddington developmental scale (Fig. 2a; Waddington *et al*., 1983). Under LDs and SDs, the SAM of Bowman(*eam10*) developed significantly faster than that of Bowman (Table S1). Significant genetic differences at single time points of dissection were only detected after Waddington stages 8 and 4 in LD and SD conditions, respectively (Fig. 2a). Under SDs, the most prominent differences between genotypes were observed in the distance between the base and the meristem (stem elongation), which was significantly larger in Bowman(*eam10*) than in Bowman at most time points (Fig. 2a). Therefore, *eam10* primarily affected stem elongation and had marginal effects on SAM development.

In barley, expression of *HvFT1* correlates with flowering time (Turner *et al*., 2005). To test whether differences in the expression of *HvFT1* could explain the observed flowering-time phenotypes, we measured expression of *HvFT1* in the different Bowman introgression lines under LDs and SDs (Fig. 1b). After 2 wk under LDs, *HvFT1* was expressed only in lines with the dominant *Ppd-H1* allele, Bowman(*Ppd-H1*) and Bowman(*Ppd-H1+eam10*). After 4 wk under SDs, *HvFT1* expression was detected in Bowman(*Ppd-H1+eam10*), but not in the other genotypes. To identify expression differences between Bowman and Bowman(*eam10*) during plant development, we analyzed *HvFT1* expression in leaf samples from plants dissected for meristem scoring under LDs and SDs (Fig. 2b). Under LDs, *HvFT1* expression was higher in Bowman(*eam10*) than in Bowman at the double-ridge stage until early stem elongation (Waddington stages 2–6; 9 and 24 DAS). Under SDs, *HvFT1* expression was detected in Bowman(*eam10*) after the beginning of stem elongation (beyond Waddington stage 3; 79 DAS), but not in Bowman. Thus, the *eam10* mutation caused an up-regulation of *HvFT1* and accelerated flowering time under both LDs and SDs. The dominant *Ppd-H1* allele in the background of *eam10* caused a stronger up-regulation of *HvFT1* than the recessive *ppd-h1* allele under both LD and SD conditions (Fig. 1b).

### *eam10* affects the expression of circadian clock homologs

**Figure 3:**
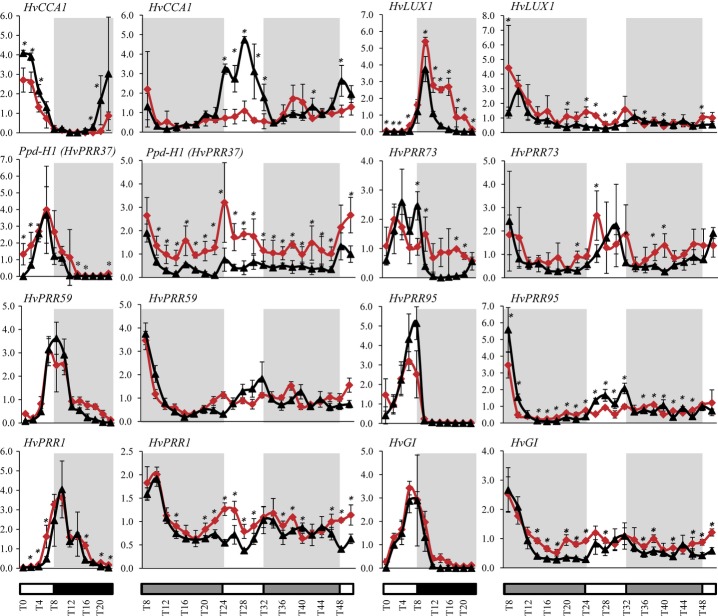
Expression patterns of main circadian clock genes in barley (*Hordeum vulgare*). Expression of *HvCCA1*, *HvLUX1, Ppd-H1* (*HvPRR37*)*, HvPRR73, HvPRR59, HvPRR95, HvPRR1*, and *HvGI* in Bowman (black lines) and Bowman(*eam10*) plants (red lines) under short-day and continuous-light conditions. White, black and grey bars indicate days, nights and subjective nights, respectively. Values represent averages of two biological and two technical replicates of expression values relative to *HvActin* ± SD. Significant differences in gene expression are indicated: *, *P* < 0.05.

Previous studies have shown that mutations in circadian clock genes caused earliness and day-neutrality in Arabidopsis and barley plants (Davis, 2002; Hazen *et al*., 2005; Onai & Ishiura, 2005; Faure *et al*., 2012; Zakhrabekova *et al*., 2012). To investigate whether the *eam10* mutation led to a disruption of clock rhythmicity, we studied diurnal (under SDs) and circadian (LL) expression of barley clock homologs, *HvCCA1*, *Ppd-H1* (*HvPRR37)*, *HvPRR73, HvPRR59, HvPRR95*, *HvPRR1*, *HvGI*, and *HvLUX1* in Bowman and Bowman(*eam10*) (Fig. 3).

Under SDs, Bowman(*eam10*) showed reduced expression of *HvCCA1* and *HvPRR73* compared with Bowman. Conversely, expression levels of *Ppd-H1* and *HvPRR1* were higher in Bowman(*eam10*) than in Bowman during the day, while the night peak of expression of *HvLUX1* and *HvPRR73* was higher in Bowman(*eam10*) than in Bowman. Expression differences observed under SDs were even more pronounced under LL. The strongest differences between Bowman and the *eam10* mutant were detected in the expression of *HvCCA1* and *Ppd-H1* under LL. Under these conditions, in Bowman(*eam10*), circadian amplitude of *HvCCA1* expression was significantly reduced during subjective days, while *Ppd-H1* was significantly up-regulated in Bowman(*eam10*) compared with Bowman at all time points. In contrast to *Ppd-H1*, expression of its homologs *HvPRR73*, *HvPRR59*, and *HvPRR95* was not strongly affected by the *eam10* mutation. The evening-expressed genes *HvPRR1* and *HvGI* were up-regulated in Bowman(*eam10*) compared with Bowman. Taken together, these results indicate that *eam10* alters the expression of barley homologs of Arabidopsis clock genes, in particular the expression of *Ppd-H1* and *HvCCA1*.

In Arabidopsis, the expression of genes implicated in the regulation of photosynthesis and photoperiod-dependent flowering is under circadian control. We therefore tested whether *eam10* changed the diurnal and circadian expression of the following genes: the photoperiod response genes *HvCO1* and *HvFT1*; the chlorophyll A/B binding protein gene *HvCABIII* from the photosynthetic pathway; and *HvCCR2*, encoding the barley ortholog of the *GYLCINE-RICH RNA-BINDING PROTEIN 7* (*GRP7*/*CCR2*), characterized as a slave (nonself-sustaining) oscillator (Schöning & Staiger, 2005; Fig. S1). Under SDs, *HvCO1* did not show differences in expression, and *HvFT1* expression was not detected in any of the two genotypes at 21 DAS. Under LL, *HvCO1* showed a small but significant up-regulation in Bowman(*eam10*) at some time points during the subjective night. Bowman(*eam10*) exhibited a strong up-regulation of *HvFT1* during the subjective day compared with Bowman. In Bowman(*eam10*), *HvCABIII* was up-regulated at nights and subjective nights, while the peak expression of *HvCCR2* was reduced under SDs. The *eam10* mutation thus affected the expression of clock output genes.

### Identification of barley *Hvlux1* as a candidate gene underlying *eam10*

To identify candidate genes underlying the *eam10* QTL, we investigated polymorphism of 133 flowering-related barley genes in Bowman and Bowman(*eam10*) using high-throughput sequencing of libraries enriched for flowering-related genes. In total, we found five SNPs between Bowman and Bowman(*eam10*) (Fig. S2). These SNPs resided within three flowering-related HarvEST unigenes, 16001, 19636, and 22370, homologous to the Arabidopsis genes *CONSTITUTIVE PHOTOMORPHOGENIC 1* (*COP1*), *LUX*, and *ABA-RESPONSIVE ELEMENT BINDING PROTEIN 2* (*ABF4*), respectively.

As reported by Druka *et al*. (2011), Bowman(*eam10*) carries introgressions on four chromosomes, 3H (single polymorphic marker), 4H (*c*. 11 cM), 6H (*c*. 15 cM), and 7H (single polymorphic marker). Using the GenomeZipper (Mayer *et al*., 2009), we mapped all three barley genes polymorphic between Bowman and Bowman(*eam10*) within the introgressed regions. The barley *LUX*-like gene designated as *HvLUX1* was located on the linkage group 3H (Hv3) at the position of POPA marker 2_1500 (171.64 cM), whereas the two other barley genes were mapped on the linkage group 6H both in the region from 85.16 to 91.79 cM. Among the three genes, only *HvLUX1* colocated with the *eam10* QTL, mapped on the distal end of the long arm of chromosome 3H below the marker ABC166 (155.85 cM; Börner *et al*., 2002) (Fig. S2).

**Figure 4:**
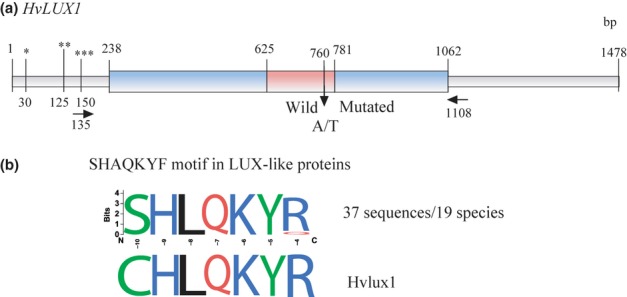
The structure of the barley (*Hordeum vulgare*) *HvLUX1* gene and the effect of a point mutation. (a) The structure of the *HvLUX1* gene and the position of a nonsynonymous single nucleotide polymorphism (SNP) in the MYB domain. The exon region is shown in blue with the region encoding the GARP family MYB domain (approximate location) highlighted in red, and the UTR or promoter regions shown in grey. PCR primers for amplification of *HvLUX1* are shown as horizontal arrows. Distances are shown in pairs of nucleotides relative to the full-length *HvLUX1* gene (NCBI accession no. AK357505). A vertical arrow indicates the position of a nonsynonymous SNP with the wild allele (A) in *HvLUX1* and the mutated allele (T) in *Hvlux1*. An evening element (*, AAATATCT) and two LUX binding site motifs (**, GATACG; ***, GATTCG) are shown in the promoter region. (b) The amino-acid mutation in the highly conserved SHLQKY(R/Q) motif in Hvlux1. An alignment of 37 SHLQKY(R/Q) motifs from 19 species (Fig. S3) is showed as a sequence logo. Green letters, polar amino-acid residues (aa); blue, basic aa; red, acidic aa; black, hydrophobic aa. The height of the letters illustrates the frequency of a corresponding aa in the alignment.

The Harvest unigene 19636 comprised only a partial coding sequence of *HvLUX1*. Therefore, we extracted its homolog Hv.20312, a full-length unigene, from NCBI GenBank (AK357505). Similarly to the Arabidopsis *LUX* gene (Hazen *et al*., 2005; Onai & Ishiura, 2005) characterized as a transcription factor (TF), the intronless *HvLUX1* gene encodes an MYB-domain-containing SHAQKYF**-**type GARP family protein of 274 amino-acid residues (Fig. 4a). An evening element (EE, AAATATCT) characteristic of clock genes expressed in the evening (e.g. Arabidopsis *TOC1* and *LUX*; Alabadí *et al*., 2001; Hazen *et al*., 2005) was found 208 bp upstream of the start codon. In addition to the EE, the promoter region of *HvLUX1* comprised two conserved LUX binding site motifs (GATACG and GATTCG) required for *LUX* autoregulation in Arabidopsis (Helfer *et al*., 2011).

Database search identified 36 LUX-like proteins from 19 plant species. Multiple alignment of these proteins revealed two highly conserved regions corresponding to the MYB and as yet uncharacterized domains (Fig. S3). The family-defining SHAQKYF motif (SHLQKY(R/Q) in the case of *LUX*-like genes) was conserved across all LUX-like sequences, thus indicating that the integrity of this motif may be critical for the function of LUX-like TFs. The mutation identified in the *LUX*-like gene from Bowman(*eam10*) changed the first amino-acid residue in this conserved motif (Fig. 4b; GenBank accession no. KC668258). Therefore, we suggested that this could lead to a disruption of LUX function and tentatively assigned *Hvlux1* as a candidate gene underlying *eam10*.

To corroborate the link between the *Hvlux1* mutation and the early flowering, we conducted a segregation analysis using 1002 BC_1_F_2_ lines derived from backcrossing Bowman(*eam10*) to Bowman. Based on sequencing analysis of early-flowering BC_1_F_2:3_ lines, we confirmed that the *Hvlux1* mutation cosegregated with the earliness phenotype.

Since the region of the *eam10* QTL, spanning *c*. 17 cM, might contain alternative candidate genes, explaining the observed phenotypes and physically linked to *Hvlux1*, we searched this region for homologs of clock and flowering genes. A barley homolog of Arabidopsis response regulator (*ARR*) clock gene (Salomé *et al*., 2006) was found *c*. 16 cM above *HvLUX1* and excluded as an alternative candidate based on the sequence analysis (Notes S1; Tables S4, S5).

### Natural diversity of *HvLUX1*

**Figure 5:**
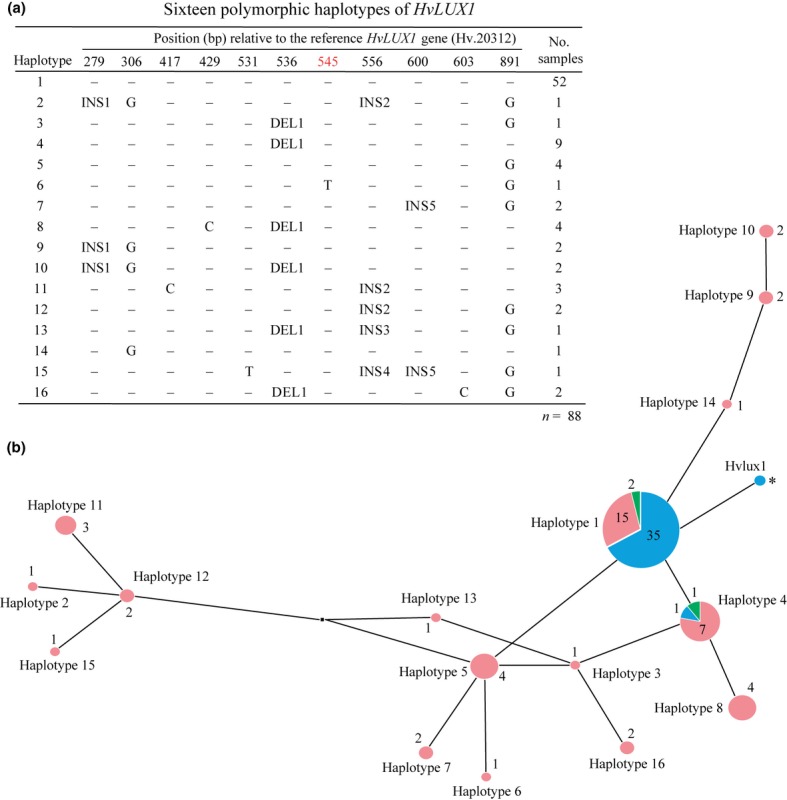
*HvLUX1* haplotypes in wild and cultivated barley (*Hordeum vulgare*). (a) Sixteen polymorphic haplotypes of *HvLUX1*. Insertions: INS1, GGAGGAGGA; INS2, ACAGCAACA; INS3, ACA; INS4, ACAGCAACAACA; INS5, GCG; deletion: DEL1, GTC. A nonsynonymous single nucleotide polymorphism (SNP) is highlighted in red. (b) Median-joining network of 16 *HvLUX1* haplotypes. Numbers at the nodes indicate the number of genotypes carrying the corresponding haplotype (out of 88 accessions). The haplotype frequency is also reflected in the relative size of a node. The color of a node corresponds to the different species: blue, *Hordeum vulgare* ssp. v*ulgare*; red, *H. vulgare* ssp. *spontaneum*; green, *H. agriocrithon*. The mutated X-ray-derived *Hvlux1* allele from Bowman is labeled with an asterisk.

Natural variation at flowering time loci in barley has been proposed to be adaptive (Jones *et al*., 2008; Comadran *et al*., 2012). To investigate natural variation in *HvLUX1*, we sequenced the full-length gene from 88 diverse wild and cultivated barley genotypes from various geographic origins. A haplotype analysis identified 16 *HvLUX1* haplotypes (Fig. 5a) distinguished by seven SNPs and six indels. The nonsynonymous SNP(545) and six in-frame indels resulted in minor changes in the amino-acid composition of HvLUX1, although none of them led to a protein truncation. The majority of the investigated genotypes, 52 samples, carried a ‘haplotype 1’ of *HvLUX1*. Diversity of *HvLUX1* was much higher in wild than in cultivated genotypes. We identified 16 haplotypes in 52 wild barley accessions, while 35 out of 36 cultivated accessions comprised a ‘haplotype 1’. In our study, mutations in *HvLUX1* did not associate with particular subspecies or place of origin (Fig. 5b; Table S2). The *Hvlux1* allele was not found in the natural population, apparently because of its artificial origin, but the pattern of its variation clearly suggests that it originated from haplotype 1.

### Phylogenetic framework of *LUX*-like genes in plants

**Figure 6:**
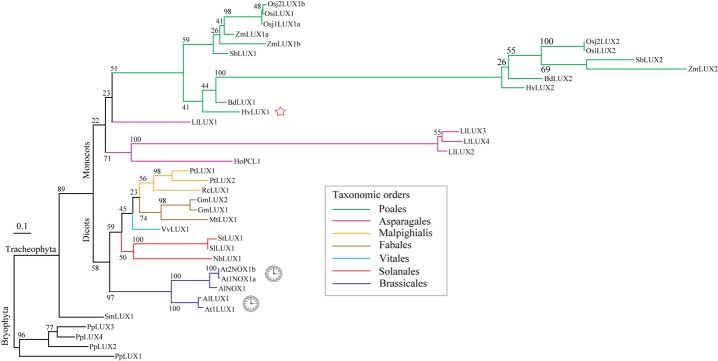
Maximum-likelihood phylogeny of LUX-like proteins in 19 plant species. Bootstrap values (%) are shown at the nodes. Clock pictograms mark Arabidopsis paralogs, LUX and NOX implicated in regulation of the circadian clock (Dai *et al*., 2011; Helfer *et al*., 2011). The LUX-like protein encoded by the gene described in this study is marked by a red star pictogram. Scale bar, 0.1 amino-acid substitutions per site. Colour of the branches indicates angiosperm orders. Protein nomenclature is as described in the Materials and Methods section.

The maximum-likelihood analysis of the LUX homologs extracted by the Blast search defined a LUX-family clade comprising Arabidopsis LUX along with the 38 LUX-like proteins from 7 monocots, 10 dicots, the spikemoss *S. moellendorffii*, and the moss *P. patens*. To infer phylogenetic relationships within the LUX family, we recalculated the phylogeny, including only members of the LUX-family clade (Fig. 6). The LUX-like proteins from the angiosperms fell into two monophyletic clades of dicots and monocots. Within these clades, distinct subclades and branches at the level of orders and families could be recognized. The topology of the Brassicales and Poales clades was indicative of independent duplications of the *LUX*-like loci in these taxa. In the *Arabidopsis* genus, such duplication apparently preceded speciation (*LUX* and *NOX/BROTHER OF LUX* genes; Hazen *et al*., 2005; Dai *et al*., 2011), whereas in monocots we tentatively ascribed it to a deeper phylogenetic level, the common ancestor of the Poaceae species (duplicates assigned as *LUX1* and *LUX2*). It is remarkable that the LUX outparalogs (*sensu* Sonnhammer & Koonin, 2002) in Poaceae significantly diverged after duplication as manifested by the distance between the LUX1 and LUX2 clades (ML distance 1.22). By contrast, the divergence between the Arabidopsis LUX-like outparalogs remained relatively low (ML distance 0.42).

**Figure 7:**
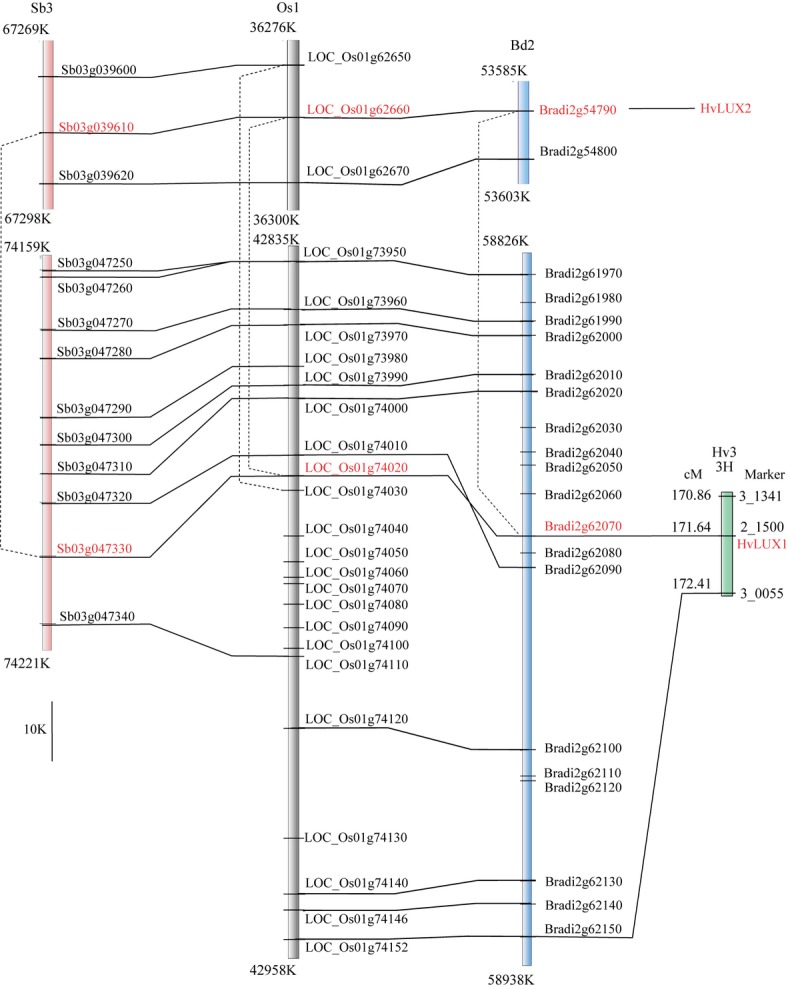
Microcollinearity in the vicinity of duplicated *LUX*-like loci in Poaceae species. Comparison of the *Sorghum bicolor* chromosome 3 (Sb3), the *Oryza sativa* ssp. *japonica* chromosome 1 (Os1), and the *Brachypodium distachyon* chromosome 2 (Bd2) was based on the microsynteny between corresponding regions on the physical maps of these species (Sb, JGI map v1.4; Os, MSU Rice Genome Annotation v7; Bd, JGI v1) as revealed by the CoGe GEvo script (http://genomevolution.org/CoGe/GEvo.pl). Location of the *Hordeum vulgare* ssp. *vulgare LUX1* locus (*HvLUX1*, as POPA marker 2_1500) is shown on the consensus genetic map (as found in the barley GenomeZipper); location of *HvLUX2* could not be established. Orthologs are connected by horizontal solid lines, outparalogs by dashed lines. *LUX*-like genes (*SbLUX1*, *Sb03 g047330*; *Osj2LUX1b*, *LOC_Os01 g7420*; *BdLUX1*, *Bradi2 g62070*; *SbLUX2*, *Sb03 g039610*; *Osj2LUX2*, *LOC_Os01 g62660*; *BdLUX2*, *Bradi2 g54790*) are highlighted in red. Scale bar, 10 kbp.

To investigate in detail the ancestral *LUX* duplication in the Poaceae, we analyzed microsynteny of the regions comprising *LUX*-like loci in Brachypodium, barley, rice, and sorghum (Fig. 7). This analysis revealed a ‘fuzzy’ homology of a *c*. 40 kbp fragment of the barley physical map (IBSC, 2012), carrying the *HvLUX1* locus, with the corresponding genomic regions of the other Poaceae species. Apparently, the order of the contigs in this region of the barley physical map awaits further refinement. Nevertheless, we could locate *HvLUX1* on the barley chromosome 3H (Hv3). *HvLUX2* was not found on either the physical or the genetic maps of barley.

The *LUX1* genes resided in syntenic regions of the chromosomes Bd2, Hv3, Os1, and Sb3 of Brachypodium, barley, rice, and sorghum, respectively, which have been shown to be orthologous (Mayer *et al*., 2011). It is noteworthy that a *c*. 10 kbp fragment comprising *BdLUX1* was inverted, while the immediately adjacent orthologous gene pairs were in a collinear order. The *LUX2* gene was found on the same chromosomes in the inverted fragment located *c*. 5.2–6.8 mbp upstream of the *LUX1* locus in all the three grass species. Several genes in the vicinity of *LUX* loci were unique to either rice or Brachypodium. This implies that gene gain and loss events occurred at these loci after speciation.

## Discussion

### Bowman(*eam10*) is a circadian clock mutant

In this study, we describe the barley mutant locus *eam10*, which accelerates flowering under LDs and, in addition, allows the plant to flower and mature under SDs. *eam10* has been isolated and described by Favret & Ryan (1966), when early cultivars had become an important goal in barley breeding programs and a large number of additional *eam* mutants were isolated in the Swedish breeding programs (Lundqvist, 2009). Recently, mutations underlying *eam8* have been located in *HvELF3* (Faure *et al*., 2012; Zakhrabekova *et al*., 2012). These mutations resulted in the disrupted expression rhythmicity of barley homologs of Arabidopsis clock genes such as *HvCCA1*, *HvPRR1* and *HvGI*, the up-regulation of genes involved in the photoperiodic pathway (e.g. *Ppd-H1*) and the acceleration of flowering independent of photoperiod. Similar effects of mutations in *ELF3*-like genes on the rhythmicity of clock genes and photoperiodic reactions have been reported in Arabidopsis, rice, pea (*Pisum sativum*), and lentils (*Lens culinaris*) (Zagotta *et al*., 1996; Saito *et al*., 2012; Undurraga *et al*., 2012; Weller *et al*., 2012; Zhao *et al*., 2012). In Bowman(*eam10*), diurnal and circadian expression patterns of clock components such as *HvCCA1*, *HvPRR95*, and *HvGI* dampened under LL conditions, thus indicating that the circadian rhythms in Bowman(*eam10*) were compromised. In both *eam8* and *eam10* mutants, disruption of circadian rhythmicity, as attested by the change in expression patterns of clock genes, accompanied acceleration of flowering irrespective of day length. As shown for Bowman(*eam8)* (Faure *et al*., 2012), early flowering of Bowman(*eam10)* was associated with an upregulation of *HvFT1* under SDs (Fig. 2b). Thus, mutations in *eam8* and *eam10* caused an induction of the long-day photoperiod pathway under noninductive SD conditions. Therefore, *eam8* and *eam10* might be part of the mechanism modulating light signal transduction from receptors to downstream components of the photoperiod pathway as has been shown for the EC genes in Arabidopsis (McWatters *et al*., 2000; Carre, 2002).

Rate of stem elongation was significantly accelerated in *eam10* mutants, whereas the timing of meristem development closely followed that of Bowman, especially at the earlier stages. In Arabidopsis, abnormally elongated hypocotyls are characteristic of a dysfunctional circadian clock, and molecular pathways underlying this phenomenon have been outlined (Niwa *et al*., 2009; Nusinow *et al*., 2011). It is tempting to speculate that while organs abnormally elongated at the early developmental stages of circadian-defective barley and Arabidopsis plants are clearly different, underlying molecular mechanisms might be similar.

### *eam10* regulates expression of *Ppd-H1*

Measurements of circadian-regulated gene expression suggested that the circadian defects in Bowman(*eam10*) were weak under SD conditions and significantly enhanced under LL in the absence of external cues. Light and temperature signals could thus drive rhythms in Bowman(*eam10*). However, under LD and SD conditions when external cues were present, *eam10* had significant effects on flowering time, apparently through a direct or indirect interaction with *Ppd-H1*. *eam10* mutant lines with the wildtype *Ppd-H1* allele flowered earlier than those with the recessive *ppd-H1* allele under LDs and SDs. This difference was most pronounced under SDs. Thus, variation at *Ppd-H1* in the background of *eam10*, affected flowering under SDs, while previously it has been postulated that *Ppd-H1* acts exclusively under LDs (Turner *et al*., 2005). The interaction between *eam10* and *Ppd-H1* was further supported by a significant up-regulation of *Ppd-H1* expression in Bowman(*eam10*) compared with Bowman under both SDs and LLs. These results suggested that *Eam10* acts as a repressor of *Ppd-H1*. Early flowering of lines carrying *Ppd-H1* and *eam10* correlated with an up-regulation of *HvFT1* under SDs (Fig. 1b), presumably caused by the up-regulation or differences in the diurnal expression pattern of *Ppd-H1* in the *eam10* background.

### *HvLUX1* is a candidate gene underlying *eam10*

The identification of a mutation in an extremely conserved region of *HvLUX1* strongly suggested *Hvlux1* as a gene underlying the *eam10* locus. The segregation analysis, the flowering and expression phenotypes, and the screening of genes in the vicinity of *eam10* solidified the link between *Hvlux1* and *eam10*, despite the possible shortcomings of using introgression lines carrying multiple genes transferred from donor germplasm. The mechanistic role of *Hvlux1* in the *eam10* flowering and expression phenotypes is yet to be discovered.

It has been shown in Arabidopsis that LUX and ELF3 together with ELF4 form the so-called ‘evening’ complex and have the same transcriptional targets (Kolmos *et al*., 2011; Nusinow *et al*., 2011; Herrero *et al*., 2012). Both LUX and ELF3 physically associate with the promoter of *PRR9* to repress its transcription (Dixon *et al*., 2011; Herrero *et al*., 2012). Up-regulation of *Ppd-H1* in *eam10* and *eam8* mutants (Faure *et al*., 2012) suggested that *HvLUX1* and *HvELF3* act in the same pathway, and that their transcriptional targets are partly conserved between Arabidopsis and barley. Interestingly, the repressive function of *eam10* was only seen for *HvPRR37*, while expression of its paralogs *HvPRR73* and *HvPRR59/95* was not strongly affected. By contrast, EC genes in Arabidopsis control expression of *PRR7* and *PRR9* (Kolmos *et al*., 2009; Herrero *et al*., 2012). Differences in the transcriptional targets of barley Eam8/Eam10 and Arabidopsis ELF3 suggest a different clock construction in barley and Arabidopsis. Bowman(*eam10*) was characterized by a down-regulation of *HvCCA1* and an up-regulation of *HvPRR1*, *HvGI*, and *HvLUX*. This is consistent with the role of Arabidopsis LUX, which activates CCA1 and represses TOC1 (PRR1), GI, and LUX itself (Hazen *et al*., 2005; Onai & Ishiura, 2005). Up-regulation of *HvLUX* in *eam10* and the presence of two conserved LUX binding site motifs (GATACG and GATTCG) in the promoter region of *HvLUX* suggested that HvLUX1 controls its own expression, as seen in Arabidopsis (Helfer *et al*., 2011).

### *LUX* duplicated independently in Poaceae and Arabidopsis

Interspecific comparison of clock genes within phylogenetic frameworks may facilitate integration of knowledge and also pinpoint dissimilar patterns of gene evolution, when direct comparisons between homologous genes from distant plant lineages should be made with greater caution. Phylogeny-supported comparison of flowering genes in grasses and Arabidopsis has identified several cases when direct orthology between members of gene families could not be established (e.g. *CDF1*, *TOE1*, *TEM*, *HAP* genes; Higgins *et al*., 2010). In the case of clock genes, such information is extremely scarce. It has been suggested that, in monocots and eudicots, genome duplication events led to the independent duplication and diversification of the *PRR* gene family (Murakami *et al*., 2007; Takata *et al*., 2010; Campoli *et al*., 2012b).

In our study, *LUX*-like genes could be traced down to mosses and apparently represent an ancient branch of the GARP transcription factor family. We found duplication of *LUX* genes in the ancestor of Poaceae (*LUX1* and *LUX2*) independent of the duplication in Arabidospis (*LUX* and *NOX*). While *LUX* paralogs in Arabidopsis retained partially redundant functions (Dai *et al*., 2011) and remained phylogenetically close, *LUX* paralogs in grasses significantly diverged before grass species radiation. This suggests that evolutionary fate of individual members of clock gene families might be different, especially in distant plant lineages.

Microsynteny around *LUX* loci in Brachypodium, rice, and sorghum revealed ancestral duplication, which is consistent with the phylogeny. However, we could not resolve whether this duplication originated from the ancient whole-genome duplication event or local translocation. Several gene loss/gain and microinversion events occurred independently in the genomes of the three grass species, while both *LUX* loci remained intact. These data implicate selection for retention of *LUX* loci in grass genomes, corroborating previous findings, which suggested preferential retention of duplicated clock genes in *Brassica rapa* (Lou *et al*., 2012).

### *HvLUX1* is under purifying selection

Structured allelic variation at several flowering- and clock-related loci has been proposed as a signature of plant adaptation to either natural or agricultural environments (Izawa, 2007; Clotault *et al*., 2012; Weller *et al*., 2012; Anwer & Davis, 2013). Allelic variation at three flowering loci, *Ppd-H1*, *HvCEN*, and *HvELF3*, associated with the geographical distribution of wild and cultivated barley habitats has been suggested to be adaptive (Jones *et al*., 2008; Comadran *et al*., 2012; Faure *et al*., 2012; Zakhrabekova *et al*., 2012).

We found that *HvLUX1* diversity is high (16 haplotypes in 88 accessions). Surprisingly, only one haplotype was prevalent in cultivars and landraces from diverse geographic origins; the diversity of *HvLUX1* was predominantly confined to wild barley (16 haplotypes in 49 accessions). Remarkably, all in-frame indels identified in several *HvLUX1* haplotypes resulted from variation in a number of microsatellite repeats. Functional and adaptive variation in the number of tandem amino-acid repeats have been demonstrated in flowering and clock genes, such as *ELF3* in Arabidopsis and *FCA* in endemic Hawaiian mint (*Stenogyne rugosa*) (Lindqvist *et al*., 2007; Undurraga *et al*., 2012). Whether variation in *HvLUX1* is functional warrants further investigation. The absence of frameshifts and predominance of a single haplotype indicate that *HvLUX1* was under purifying selection. While barley plants with nonfunctional *Hvelf3* might have selective advantage in certain environments, integrity of *HvLUX1* seems to be under functional constraints.

### Conclusion

Isolation and characterization of *Hvlux1* advance our understanding of the photoperiod response pathway in temperate cereals. The mutation in *HvLUX1* is linked to *eam10*, which caused early flowering independent of the photoperiod. *Hvlux1* was associated with similar, but weaker, molecular and flowering phenotypes than *Hvelf3*. It thus represents an interesting resource for modulating photoperiod response and ultimately adaptation in barley. Revealed patterns of diversification of duplicated LUX loci and their natural variation offer some insight into a number of questions regarding the differential fate of duplicated genes in different plant lineages and the impact of the circadian clock components on adaptation in natural populations.

## Supporting Information

**Fig. S1** Expression patterns of clock output genes. **Fig. S2** Location of genes and SNPs distinguishing between Bowman and Bowman(*eam10*) on barley linkage groups. **Fig. S3** Multiple alignment of LUX-like protein sequences from 19 plant species. **Table S1** ANOVA for meristem development and gene expression differences **Table S2** Accessions used for the resequencing of *HvLUX1* **Table S3** Barley flowering-related genes selected for targeted enrichment **Table S4** PCR primers specific for the barley *ARR*-like gene **Methods S1** Preparation of TruSeq libraries. **Methods S2** Read processing workflow using the Galaxy server (http://galaxy.wur.nl). **Methods S3** Polymorphism filtering workflow. **Methods S4** Permanent web links to the detailed results of the synteny analysis in the vicinity of *LUX* genes in three Poaceae species. **Notes S1** Sequencing of the barley *ARR*-like gene.

**Table S5** Results of the gene ontology analysis of Brachypodium genes surrounding the *LUX* locus

## References

[b1] Aird D, Ross MG, Chen WS, Danielsson M, Fennell T, Russ C, Gnirke A (2011). Analyzing and minimizing PCR amplification bias in Illumina sequencing libraries. Genome Biology.

[b2] Alabadí D, Oyama T, Yanovsky MJ, Harmon FG, Mas P, Kay SA (2001). Reciprocal regulation between *TOC1* and *LHY/CCA1* within the *Arabidopsis* circadian clock. Science.

[b3] Anwer MU, Davis SJ (2013). An overview of natural variation studies in the *Arabidopsis thaliana* circadian clock. Seminars in Cell & Developmental Biology.

[b4] Badr A, Mueller K, Schaefer-Pregl R, El-Rabey H, Efgen S, Ibrahim HH, Pozzi C, Rohde W, Salamini F (2000). On the origin and domestication history of barley (*Hordeum vulgare*. Molecular Biology and Evolution.

[b5] Beales J, Turner A, Griffiths S, Snape JW, Laurie DA (2007). A *pseudo-response regulator* is misexpressed in the photoperiod insensitive *Ppd-D1a* mutant of wheat (*Triticum aestivum* L.). Theoretical and Applied Genetics.

[b6] Börner A, Buck-Sorlin GH, Hayes PM, Malyshev S, Korzun V (2002). Molecular mapping of major genes and quantitative trait loci determining flowering time in response to photoperiod in barley. Plant Breeding.

[b7] Campoli C, Drosse B, Searle I, Coupland G, von Korff M (2012a). Functional characterisation of *HvCO1*, the barley (*Hordeum vulgare*) flowering time ortholog of *CONSTANS*. Plant Journal.

[b8] Campoli C, Shtaya M, Davis SJ, von Korff M (2012b). Expression conservation within the circadian clock of a monocot: natural variation at barley *Ppd-H1* affects circadian expression of flowering time genes, but not clock orthologs. BMC Plant Biology.

[b9] Carre IA (2002). *ELF3*: a circadian safeguard to buffer effects of light. Trends in Plant Science.

[b10] Close TJ, Bhat PR, Lonardi S, Wu Y, Rostoks N, Ramsay L, Druka A, Stein N, Svensson JT, Wanamaker S (2009). Development and implementation of high-throughput SNP genotyping in barley. BMC Genomics.

[b11] Clotault J, Thuillet AC, Buiron M, De Mita S, Couderc M, Haussmann BIG, Mariac C, Vigouroux Y (2012). Evolutionary history of pearl millet (*Pennisetum glaucum* (L.) R. Br.) and selection on flowering genes since its domestication. Molecular Biology and Evolution.

[b12] Comadran J, Kilian B, Russell J, Ramsay L, Stein N, Ganal M, Shaw P, Bayer M, Thomas W, Marshall D (2012). Natural variation in a homolog of *Antirrhinum CENTRORADIALIS* contributed to spring growth habit and environmental adaptation in cultivated barley. Nature Genetics.

[b13] Crooks GE, Hon G, Chandonia JM, Brenner SE (2004). WebLogo: a sequence logo generator. Genome Research.

[b14] Dai S, Wei X, Pei L, Thompson RL, Liu Y, Heard JE, Ruff TG, Beachy RN (2011). BROTHER OF LUX ARRHYTHMO is a component of the *Arabidopsis* circadian clock. Plant Cell.

[b15] Davis SJ (2002). Photoperiodism: the coincidental perception of the season. Current Biology.

[b16] DePristo MA, Banks E, Poplin R, Garimella KV, Maquire JR, Hartl C, Philippakis AA, del Angel G, Rivas MA, Hanna M (2011). A framework for variation discovery and genotyping using next-generation DNA sequencing data. Nature Genetics.

[b17] Dixon LE, Knox K, Kozma-Bogmar L, Southern MM, Pokhilko A, Millar AJ (2011). Temporal repression of core circadian genes is mediated through EARLY FLOWERING 3 in *Arabidopsis*. Current Biology.

[b18] Druka A, Franckowiak J, Lundqvist U, Bonar N, Alexander J, Houston K, Radovic S, Shahinnia F, Vendramin V, Morgante M (2011). Genetic dissection of barley morphology and development. Plant Physiology.

[b19] Ehrenreich IM, Hanzawa Y, Chou L, Roe JL, Kover PX, Purugganan MD (2009). Candidate gene association mapping of Arabidopsis flowering time. Genetics.

[b20] Faure S, Turner AS, Gruszka D, Christodoulou V, Davis SJ, von Korff M, Laurie DA (2012). Mutation at the circadian clock gene *EARLY MATURITY 8* adapts domesticated barley (*Hordeum vulgare*) to short growing seasons. Proceedings of the National Academy of Sciences, USA.

[b21] Favret EA, Ryan GS (1966). New useful mutants in plant breeding. Mutations in Plant Breeding.

[b22] Gallagher LW, Franckowiak JD (1997). Description of Stock number BGS130. Barley Genetics Newsletter.

[b23] Gallagher LW, Soliman KM, Vivar H (1991). Interactions among loci conferring photoperiod insensitivity for heading time in spring barley. Crop Science.

[b24] Hall TA (1999). BioEdit: a user-friendly biological sequence alignment editor and analysis program for Windows 95/98/NT. Nucleic Acids Symposium Series.

[b25] Hazen SP, Schultz TF, Pruneda-Paz JL, Borevitz JO, Ecker JR, Kay SA (2005). *LUX ARRHYTHMO* encodes a Myb domain protein essential for circadian rhythms. Proceedings of the National Academy of Sciences, USA.

[b26] Helfer A, Nusinow DA, Chow BY, Gehrke AR, Bulyk ML, Kay SA (2011). *LUX ARRHYTHMO* encodes a nighttime repressor of circadian gene expression in the *Arabidopsis* core clock. Current Biology.

[b27] Herrero E, Kolmos E, Bujdoso N, Yuan Y, Wang M, Berns MC, Uhlworm H, Coupland G, Saini R, Jaskolski M (2012). EARLY FLOWERING4 recruitment of EARLY FLOWERING3 in the nucleus sustains the *Arabidopsis* circadian clock. Plant Cell.

[b28] Higgins JA, Bailey PC, Laurie DA (2010). Comparative genomics of flowering time pathways using *Brachypodium distachyon* as a model for the temperate grasses. PLoS ONE.

[b29] Huang W, Pérez-García P, Pokhilko A, Millar AJ, Antoshechkin I, Riechmann JL, Mas P (2012). Mapping the core of the *Arabidopsis* circadian clock defines the network structure of the oscillator. Science.

[b30] Hübner S, Höffken M, Oren E, Haseneyer G, Stein N, Graner A, Schmid K, Fridman E (2009). Strong correlation of the population structure of wild barley (*Hordeum spontaneum*) across Israel with temperature and precipitation variation. Molecular Ecology.

[b31] Huson DH, Bryant D (2006). Application of phylogenetic networks in evolutionary studies. Molecular Biology and Evolution.

[b32] Huson DH, Scornavacca C (2012). Dendroscope 3: an interactive tool for rooted phylogenetic trees and networks. Systematic Biology.

[b33] International Barley Sequencing Consortium (IBSC) (2012). A physical, genetic and functional sequence assembly of the barley genome. Nature.

[b34] Izawa T (2007). Adaptation of flowering-time by natural and artificial selection in *Arabidopsis* and rice. Journal of Experimental Botany.

[b35] Jones H, Leigh FJ, Mackay I, Bower MA, Smith LMJ, Charles MP, Jones G, Jones MK, Brown TA, Powell W (2008). Population-based resequencing reveals that the flowering time adaptation of cultivated barley originated east of the Fertile Crescent. Molecular Biology and Evolution.

[b36] Katoh K, Toh H (2008). Recent developments in the MAFFT multiple sequence alignment program. Briefings in Bioinformatics.

[b37] Kolmos E, Herrero E, Bujdoso N, Millar AJ, Tóth R, Gyula P, Nagy F, Davis SJ (2011). A reduced-function allele reveals that *EARLY FLOWERING3* repressive action on the circadian clock is modulated by phytochrome signals in *Arabidopsis*. Plant Cell.

[b38] Kolmos E, Nowak M, Werner M, Fischer K, Schwarz G, Mathews S, Schoof H, Nagy F, Bujnicki JM, Davis SJ (2009). Integrating ELF4 into the circadian system through combined structural and functional studies. HFSP Journal.

[b39] von Korff M, Léon J, Pillen K (2010). Detection of epistatic interactions between exotic alleles introgressed from wild barley (*H. vulgare* ssp. *spontaneum*. TAG. Theoretical and Applied Genetics.

[b41] von Korff M, Wang H, Léon J, Pillen K (2006). AB-QTL analysis in spring barley: II. Detection of favourable exotic alleles for agronomic traits introgressed from wild barley (*H. vulgare* ssp. *spontaneum*. Theoretical and Applied Genetics.

[b43] Laurie DA, Pratchett N, Bezant JH, Snape JW (1995). RFLP mapping of five major genes and eight quantitative trait loci controlling flowering time in a winter × spring barley *Hordeum vulgare* L. cross. Genome.

[b44] Li H, Durbin R (2009a). Fast and accurate short read alignment with Burrows–Wheeler transform. Bioinformatics.

[b45] Li H, Handsaker B, Wysoker A, Fennell T, Ruan J, Homer N, Marth G, Abecasis G, Durbin R (2009b). The Sequence Alignment/Map format and SAM tools. Bioinformatics.

[b46] Lindqvist C, Laakkonen L, Albert VA (2007). Polyglutamine variation in a flowering time protein correlates with island age in a Hawaiian plant radiation. BMC Evolutionary Biology.

[b47] Lou P, Wu J, Cheng F, Cressman LG, Wang X, McClung CR (2012). Preferential retention of circadian clock genes during diploidization following whole genome triplication in *Brassica rapa*. Plant Cell.

[b48] Lundqvist U, Shu QY (2009). Eighty years of Scandinavian barley mutation genetics and breeding. Induced Plant Mutations in the Genomics Era.

[b49] Lyons E, Freeling M (2008). How to usefully compare homologous plant genes and chromosomes as DNA sequences. Plant Journal.

[b50] Mayer KF, Martis M, Hedley PE, Simková H, Liu H, Morris JA, Steuernagel B, Taudien S, Roessner S, Gundlach H (2011). Unlocking the barley genome by chromosomal and comparative genomics. Plant Cell.

[b51] Mayer KF, Taudien S, Martis M, Simkova H, Suchankova P, Gundlach H, Wicker T, Petzold A, Felder M, Steuernagel B (2009). Gene content and virtual gene order of barley chromosome 1H. Plant Physiology.

[b52] McWatters HG, Bastow RM, Hall A, Millar AJ (2000). The *ELF3 zeitnehmer* regulates light signalling to the circadian clock. Nature.

[b53] Murakami M, Tago Y, Yamashino T, Mizuno T (2007). Comparative overviews of clock-associated genes of *Arabidopsis thaliana* and *Oryza sativa*. Plant and Cell Physiology.

[b54] Murphy RL, Klein RR, Morishige DT, Brady JA, Rooney WL, Miller FR, Dugas DV, Klein PE, Mullet JE (2011). Coincident light and clock regulation of *pseudoresponse regulator protein 37**PRR37*) controls photoperiodic flowering in sorghum. Proceedings of the National Academy of Sciences, USA.

[b55] Nagel DH, Kay SA (2012). Complexity in the wiring and regulation of plant circadian networks. Current Biology.

[b56] Niwa Y, Takafumi Y, Takeshi M (2009). The circadian clock regulates the photoperiodic response of hypocotyl elongation through a coincidence mechanism in *Arabidopsis thaliana*. Plant and Cell Physiology.

[b57] Nusinow DA, Helfer A, Hamilton EE, King JJ, Imaizumi T, Schultz TF, Farré EM, Kay SA (2011). The ELF4-ELF3-LUX complex links the circadian clock to diurnal control of hypocotyl growth. Nature.

[b58] Onai K, Ishiura M (2005). *PHYTOCLOCK 1* encoding a novel GARP protein essential for the *Arabidopsis* circadian clock. Genes to Cells.

[b59] Pokhilko A, Fernández AP, Edwards KD, Southern MM, Halliday KJ, Millar AJ (2012). The clock gene circuit in *Arabidopsis* includes a repressilator with additional feedback loops. Molecular Systems Biology.

[b60] Quevillon E, Silventoinen V, Pillai S, Harte N, Mulder N, Apweiler R, Lopez R (2005). InterProScan: protein domains identifier. Nucleic Acids Research.

[b61] Saito H, Ogiso-Tanaka E, Okumoto Y, Yoshitake Y, Izumi H, Yokoo T, Matsubara K, Hori K, Yano M, Inoue H (2012). *Ef7* encodes an ELF3-like protein and promotes rice flowering by negatively regulating the floral repressor gene *Ghd7* under both short- and long-day conditions. Plant and Cell Physiology.

[b75] Salomé PA, To JP, Kieber JJ, McClung CR (2006). *Arabidopsis* response regulators ARR3 and ARR4 play cytokinin-independent roles in the control of circadian period. Plant Cell.

[b62] SAS Institute (2009). The SAS system for Windows, release 9.1.3.

[b63] Schöning JC, Staiger D (2005). At the pulse of time: protein interactions determine the pace of circadian clocks. FEBS Letters.

[b64] Sonnhammer ELL, Koonin EV (2002). Orthology, paralogy and proposed classification for paralog subtypes. Trends in Genetics.

[b65] Stamatakis A, Hoover P, Rougemont J (2008). A rapid bootstrap algorithm for the RAxML Web servers. Systematic Biology.

[b66] Takata N, Saito S, Saito CT, Uemura M (2010). Phylogenetic footprint of the plant clock system in angiosperms: evolutionary processes of pseudo-response regulators. BMC Evolutionary Biology.

[b67] Turner A, Beales J, Faure S, Dunford RP, Laurie DA (2005). The pseudo-response regulator *Ppd-H1* provides adaptation to photoperiod in barley. Science.

[b68] Undurraga SF, Press MO, Legendre M, Bujdoso N, Bale J, Wang H, Davis SJ, Verstrepen KJ, Queitsch C (2012). Background-dependent effects of polyglutamine variation in the *Arabidopsis thaliana* gene *ELF3*. Proceedings of the National Academy of Sciences, USA.

[b69] Waddington SR, Cartwright PM, Wall PC (1983). A quantitative scale of spike initial and pistil development in barley and wheat. Annals of Botany.

[b70] Wang G, Schmalenbach I, von Korff M, Léon J, Kilian B, Rode J, Pillen K (2010). Association of barley photoperiod and vernalization genes with QTLs for flowering time and agronomic traits in a DH-population and a set of wild barley introgression lines. Theoretical and Applied Genetics.

[b71] Weller JL, Liew LC, Hecht VF, Rajandran V, Laurie RE, Ridge S, Wenden B, Vander Schoor JK, Jaminon O, Blassiau C (2012). A conserved molecular basis for photoperiod adaptation in two temperate legumes. Proceedings of the National Academy of Sciences, USA.

[b72] Zagotta MT, Hicks KA, Jacobs CI, Young JC, Hangarter RP, Meeks-Wagner DR (1996). The *Arabidopsis ELF3* gene regulates vegetative photomorphogenesis and the photoperiodic induction of flowering. Plant Journal.

[b73] Zakhrabekova S, Gough SP, Braumann I, Müller AH, Lundqvist J, Ahmann K, Dockter C, Matyszczak I, Kurowska M, Druka A (2012). Induced mutations in circadian clock regulator *Mat-a* facilitated short-season adaptation and range extension in cultivated barley. Proceedings of the National Academy of Sciences, USA.

[b74] Zhao J, Huang X, Ouyang X, Chen W, Du A, Zhu L, Wang S, Deng XW, Li S (2012). *OsELF3-1*, an ortholog of *Arabidopsis EARLY FLOWERING 3*, regulates rice circadian rhythm and photoperiodic flowering. PLoS ONE.

