# Supplementary material for: HvLUX1 is a candidate gene underlying the early maturity 10 locus in barley: phylogeny, diversity, and interactions with the circadian clock and photoperiodic pathways
Source: New Phytol. 2013 Jun 3;199(4):1045–59. doi: 10.1111/nph.12346 (PMC3902989; doi:10.1111/nph.12346)
Supplement: Supplementary file 1 — Fig. S1 Expression patterns of clock output genes. Fig. S2 Location of genes and SNPs distinguishing between Bowman and Bowman(eam10) on barley linkage groups. Fig. S3 Multiple alignment of LUX-like protein sequences from 19 plant species. Table S1 ANOVA for meristem development and gene expression differences Table S2 Accessions used for the resequencing of HvLUX1 Table S3 Barley flowering-related genes selected for targeted enrichment Table S4 PCR primers specific for the barley ARR-like gene Methods S1 Preparation of TruSeq libraries. Methods S2 Read processing workflow using the Galaxy server (http://galaxy.wur.nl). Methods S3 Polymorphism filtering workflow. Methods S4 Permanent web links to the detailed results of the synteny analysis in the vicinity of LUX genes in three Poaceae species. Notes S1 Sequencing of the barley ARR-like gene. [file nph0199-1045-sd1.docx]

**Supporting Information Figs S1–S3, Tables S1–S5, Notes S1, Methods S1–S4**

**Supporting Figures**


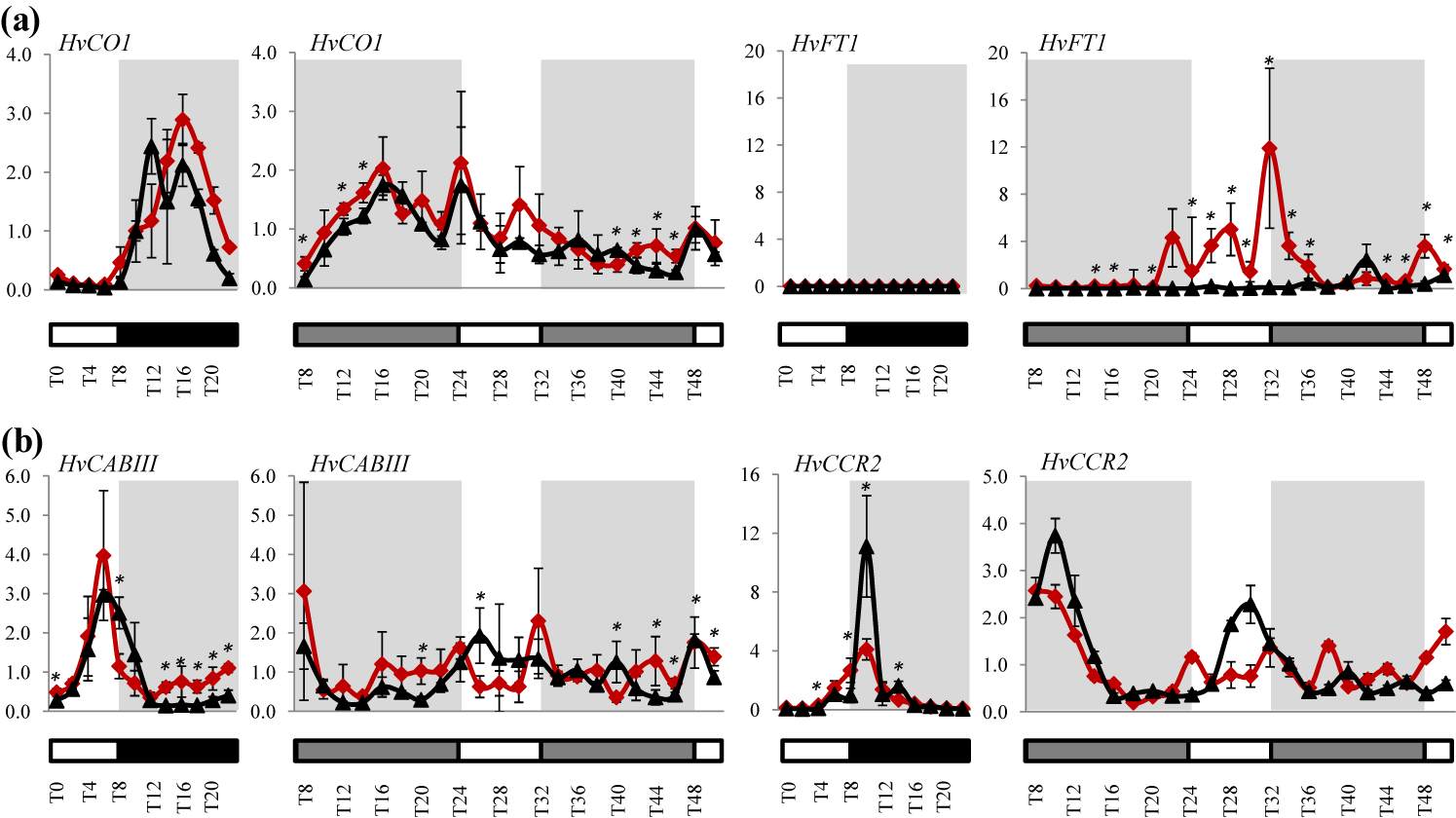


**Fig. S1** Expression patterns of clock outputs genes. (a) Photoperiodic flowering gene (*HvCO1*, *HvFT1*) and (b) clock output gene (*HvCABIII, HvCCR2*) expression in Bowman (black lines) and Bowman(*eam10)* plants (red lines) under short day (SD) and continuous light conditions. *HvFT1* expression was not detected under SDs. White, black and grey bars indicate, respectively, days, nights, and subjective nights. Values represent average of two biological and two technical replicates of expression values relative to *HvActin* plus/minus standard deviation. Significant differences in gene expression are indicated by asterisks (*, P < 0.05).

**
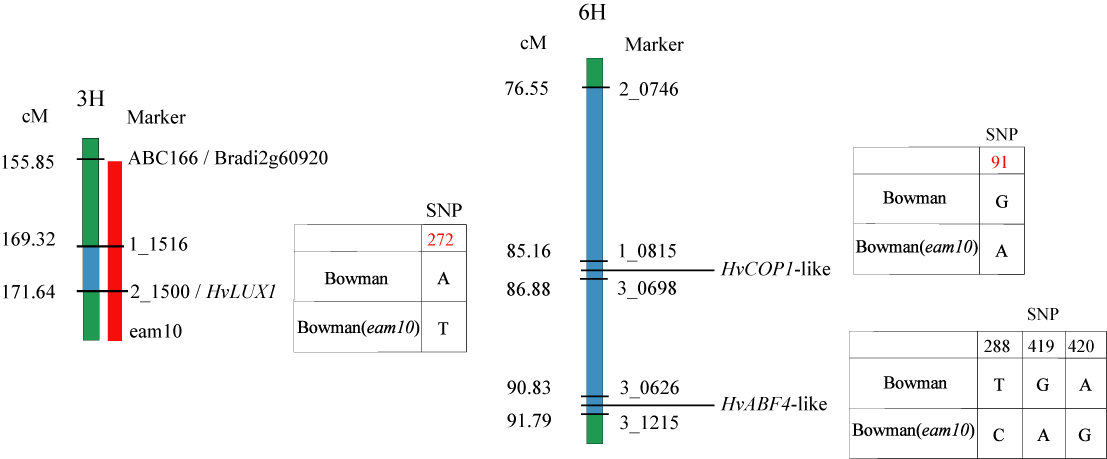
**

**Fig. S2** Location of genes and SNPs distinguishing between Bowman and Bowman(*eam10*) on barley (*Hordeum vulgare*) linkage groups. Locations of genes are shown on the barley consensus genetic map used as a framework for barley GenomeZipper. Introgressed regions are shown by blue boxes, Bowman background by green boxes. Polymorphic SNPs are shown alongside the genes. The SNP positions are given in pairs of nucleotides relative to the reference Harvest 35 unigenes 16001, 19636, and 22370 corresponding to *HvCOP1*-like, *HvLUX1*, and *HvABF4*-like genes, respectively. Positions of non-synonymous SNPs are highlighted in red. Position of the *eam10* QTL is shown by a red box.

**
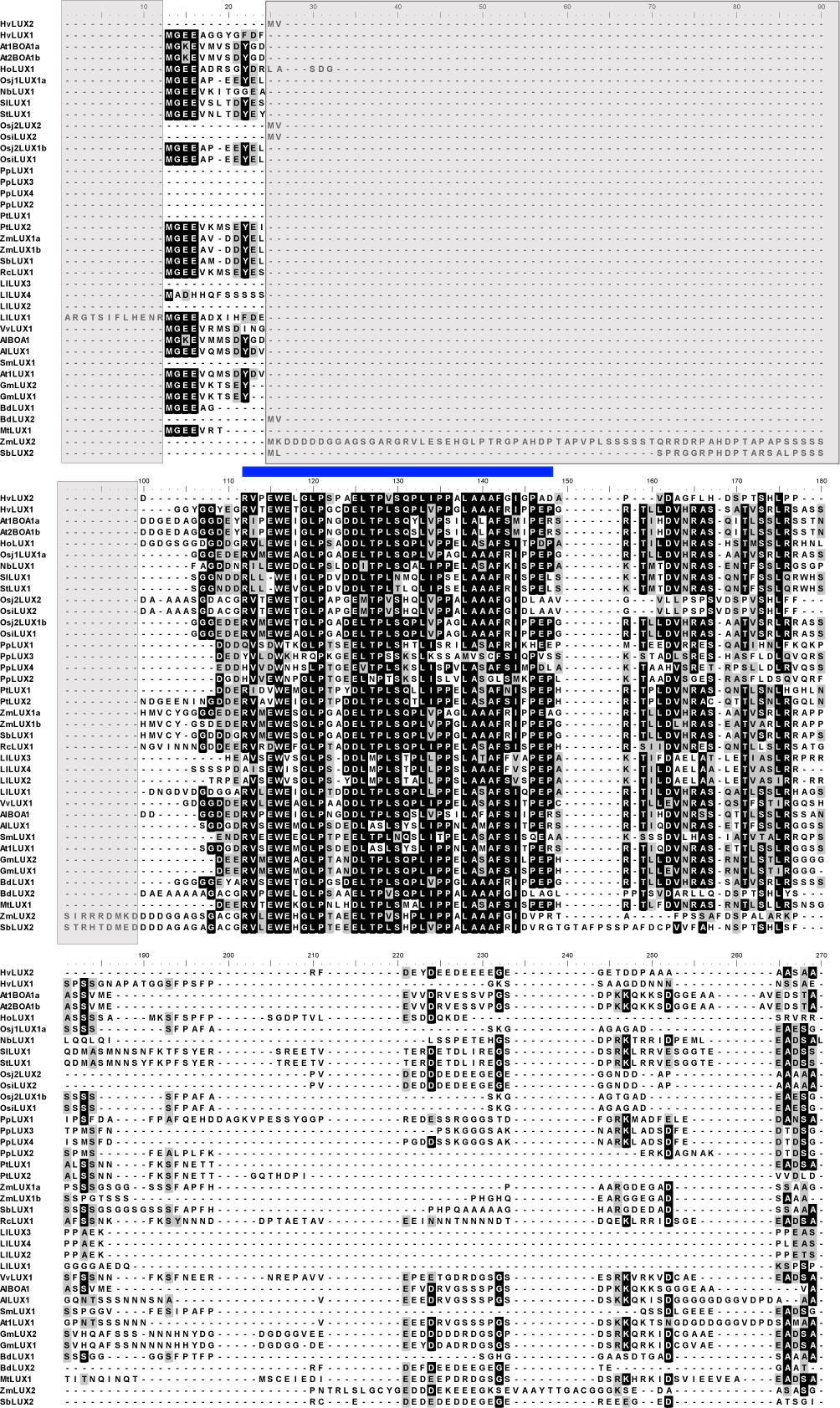
**

**
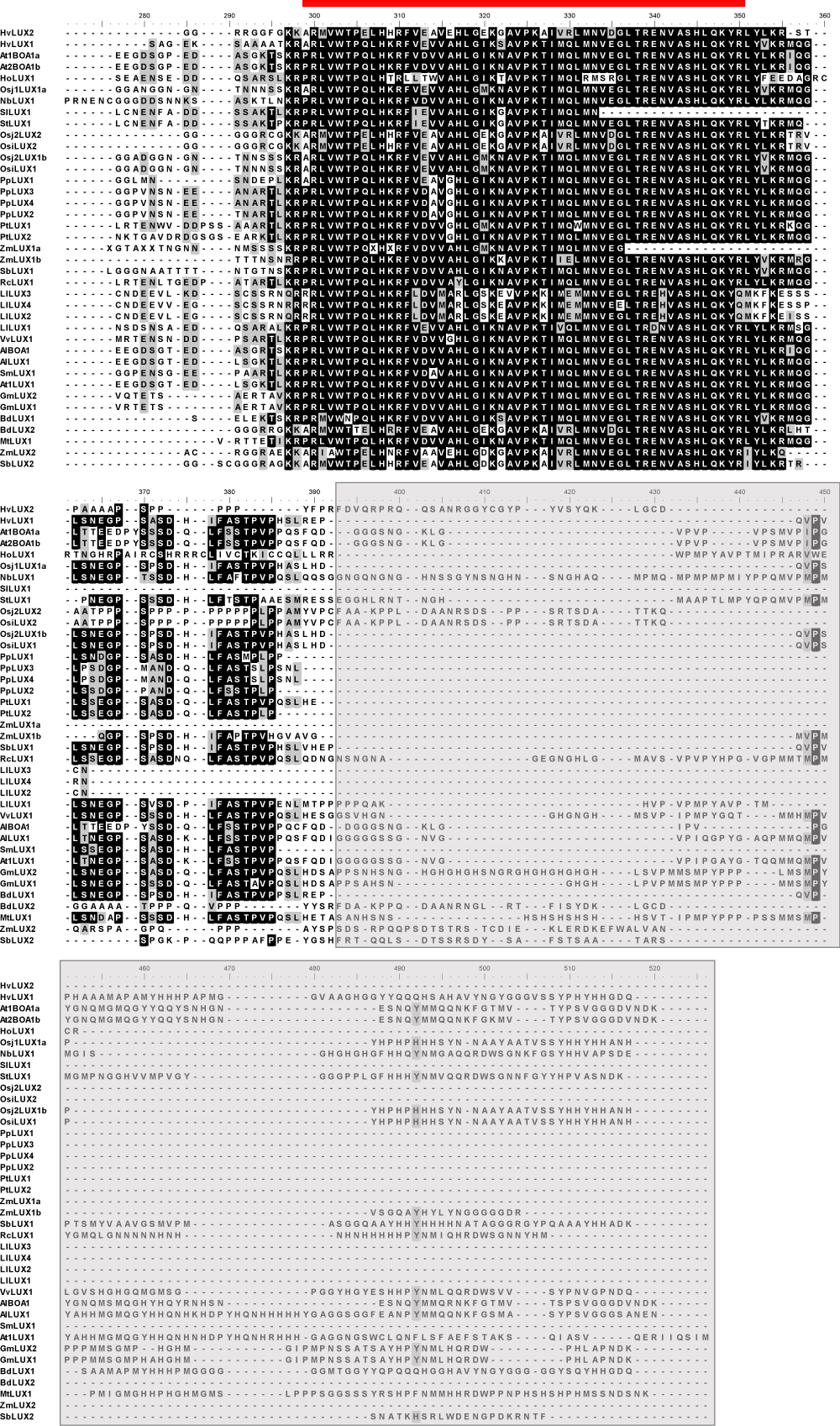
**

**Fig. S3** Multiple alignment of LUX-like protein sequences from 19 plant species. Amino-acid residues identical in all aligned sequences are shaded black, similar grey (threshold for shading 40%). Visually misaligned regions excluded from the alignment for the phylogeny reconstruction are indicated by semitransparent grey blocks. For the nomenclature of individual protein names­_­_ refer to Material and Methods. MYB and as yet undescribed conserved domains are shown, respectively, by red and blue bars.

**Supporting tables**

**Table S1** ANOVA for meristem development and gene expression differences

**(a)**

| Factor | Meristem LD | | Meristem SD | | Stem elongation | |
| --- | --- | --- | --- | --- | --- | --- |
|  | MS | R2 | MS | R2 | MS | R2 |
| Genotype (G)  Time point (T) | 10*** | 1 | 2*** | 2 | 48*** | 3 |
|  | 52*** | 97 | 9*** | 93 | 122*** | 86 |
| Biol. replicate | 0 | 0 | 0 | 0 | 1 | 0 |
| G*T | 0 | 1 | 0 | 2 | 10*** | 7 |

**(b)**

| Factor | *HvCCA1* | | *HvLux1* | | *Ppd-H1* | | *HvPRR73* | | *HvPRR59* | | *HvPRR95* | | *HvPRR1* | | *HvGI* | | *HvCO1* | | *HvCCR2* | | *HvCABIII* | |
| --- | --- | --- | --- | --- | --- | --- | --- | --- | --- | --- | --- | --- | --- | --- | --- | --- | --- | --- | --- | --- | --- | --- |
|  | MS | R2 | MS | R2 | MS | R2 | MS | R2 | MS | R2 | MS | R2 | MS | R2 | MS | R2 | MS | R2 | MS | R2 | MS | R2 |
| Genotype (G) | 11*** | 6 | 19*** | 10 | 6** | 3 | 2* | 3 | 0 | 0 | 1 | 0 | 1* | 1 | 1 | 0 | 1 | 1 | 6** | 1 | 1* | 1 |
| Time point (T) | 12*** | 70 | 14*** | 78 | 12*** | 68 | 3*** | 51 | 11*** | 63 | 17*** | 83 | 12*** | 85 | 9*** | 79 | 6*** | 73 | 35*** | 72 | 7*** | 73 |
| Biol. replicate | 2 | 1 | 0 | 0 | 4* | 2 | 0 | 0 | 0 | 0 | 0 | 0 | 0 | 0 | 0 | 0 | 0 | 0 | 0 | 0 | 0 | 0 |
| G*T | 1* | 6 | 2*** | 9 | 1 | 4 | 1** | 15 | 0 | 2 | 2** | 8 | 0 | 3 | 0 | 2 | 1** | 9 | 9*** | 18 | 1** | 9 |

**(c)**

| Factor | *HvCCA1* | | *HvLux1* | | *Ppd-H1* | | *HvPRR73* | | *HvPRR59* | | *HvPRR95* | | *HvPRR1* | | *HvGI* | | *HvCO1* | | *HvFT1* | | *HvCCR2* | | *HvCABIII* | |
| --- | --- | --- | --- | --- | --- | --- | --- | --- | --- | --- | --- | --- | --- | --- | --- | --- | --- | --- | --- | --- | --- | --- | --- | --- |
|  | MS | R2 | MS | R2 | MS | R2 | MS | R2 | MS | R2 | MS | R2 | MS | R2 | MS | R2 | MS | R2 | MS | R2 | MS | R2 | MS | R2 |
| Genotype (G) | 14*** | 8 | 9*** | 6 | 58*** | 31 | 5*** | 4 | 0 | 0 | 2* | 1 | 3*** | 8 | 4*** | 5 | 2* | 3 | 139*** | 12 | 0 | 1 | 2* | 1 |
| Time point (T) | 4** | 42 | 4*** | 52 | 3*** | 34 | 2*** | 36 | 4*** | 82 | 6*** | 80 | 1*** | 69 | 3*** | 74 | 2*** | 61 | 13*** | 24 | 4*** | 12 | 2*** | 36 |
| Biol. replicate | 3** | 2 | 0 | 0 | 2** | 2 | 0 | 0 | 0 | 0 | 0 | 0 | 0 | 0 | 0 | 0 | 1 | 1 | 5 | 1 | 0 | 1 | 0 | 0 |
| G*T | 3*** | 31 | 1*** | 13 | 1** | 7 | 1 | 12 | 0** | 10 | 1*** | 11 | 0*** | 9 | 0*** | 6 | 0 | 5 | 14*** | 26 | 1** | 12 | 1** | 16 |

Analysis of variance for **(a)** meristem development and gene expression under SD **(b)** and LL **(c)** using the factors genotype (Bowman, Bowman(*eam10*)), time point, and biological replicate. Significant effects are indicated by asterisks (*, P < 0.05; **, P < 0.01; ***, P < 0.001). MS = Means squares, R2 = proportion of the total phenotypic variance explained by the factor

**Table S2** Accessions used for the re-sequencing of HvLUX1

| *Hordeum* species | Genotype* | Status | Growth habit | Origin | *HvLUX1* haplotype |
| --- | --- | --- | --- | --- | --- |
| *vulgare* ssp. *vulgare* | Acsad | cultivar | spring | Jordan | 1 |
|  | Barke | cultivar | spring | Germany | 1 |
|  | Morex | cultivar | spring | USA | 1 |
|  | Mutah | cultivar | spring | Jordan | 1 |
|  | Rum | cultivar | spring | Jordan | 1 |
|  | Scarlett | cultivar | spring | Germany | 1 |
|  | Steptoe | cultivar | spring | USA | 1 |
|  | Yarmouk | cultivar | spring | Jordan | 1 |
|  | ER/Apm | cultivar | spring | North Africa | 1 |
|  | LR521 | cultivar | spring | Ethiopia | 1 |
|  | LR1043 | cultivar | spring | Iran | 4 |
|  | LR87 | landrace | spring | Tunisia | 1 |
|  | LR761 | landrace | spring | Algeria | 1 |
|  | LR871 | cultivar | spring | Egypt | 1 |
|  | LR1897 | cultivar | spring | Jordan | 1 |
|  | G419 | landrace | n.d. | Middle Asia | 1 |
|  | G423 | landrace | n.d. | Ethiopia | 1 |
|  | G434 | landrace | n.d. | Ethiopia | 1 |
|  | G400 | landrace | spring | Egypt | 1 |
|  | G440 | landrace | spring | Yemen | 1 |
|  | G1559 | cultivar | spring | Ethiopia | 1 |
|  | G1560 | cultivar | spring | n.d. | 1 |
|  | FT395 | cultivar | spring | Italy | 1 |
|  | FT414 | landrace | spring | Yemen | 1 |
|  | FT438 | cultivar | winter | Syria | 1 |
|  | FT439 | cultivar | winter | Syria | 1 |
|  | FT440 | cultivar | spring | Australia | 1 |
|  | FT441 | cultivar | spring | Australia | 1 |
|  | FT442 | cultivar | spring | Germany | 1 |
|  | FT443 | cultivar | spring | Germany | 1 |
|  | B1K-55-01 | landrace | n.d. | Israel | 1 |
|  | B1K-55-02 | landrace | n.d. | Israel | 1 |
|  | B1K-55-06 | landrace | spring | Israel | 1 |
|  | B1K-70-01 | cultivar | spring | Israel | 1 |
|  | B1K-70-02 | cultivar | spring | Israel | 1 |
|  | WI2297 | cultivar | spring | Australia | 1 |
| *vulgare* ssp. *spontaneum* | FT231 | wild | winter | Iraq | 1 |
|  | FT232 | wild | winter | Iraq | 4 |
|  | HID10 | wild | n.d. | Iraq | 1 |
|  | HID21 | wild | n.d. | Iran | 10 |
|  | HID46 | wild | n.d. | Iran | 2 |
|  | HID52 | wild | n.d. | Iran | 1 |
|  | HID54 | wild | n.d. | Turkey | 3 |
|  | HID257 | wild | n.d. | Israel | 5 |
|  | HID309 | wild | n.d. | Iran | 1 |
|  | HID330-1 | wild | n.d. | Former Soviet Union | 1 |
|  | HID334-1 | wild | n.d. | Former Soviet Union | 6 |
|  | HID376-2 | wild | n.d. | Israel | 4 |
|  | HID377-1 | wild | n.d. | Israel | 1 |
|  | HID377-2 | wild | n.d. | Israel | 1 |
|  | B1K-24-05 | wild | n.d. | Israel | 1 |
|  | B1K-25-01 | wild | n.d. | Israel | 16 |
|  | B1K-25-05 | wild | n.d. | Israel | 16 |
|  | B1K-26-04 | wild | n.d. | Israel | 13 |
|  | B1K-26-08 | wild | n.d. | Israel | 4 |
|  | B1K-26-11 | wild | n.d. | Israel | 5 |
|  | B1K-29-20 | wild | n.d. | Israel | 4 |
|  | B1K-30-13 | wild | n.d. | Israel | 11 |
|  | B1K-31-05 | wild | n.d. | Israel | 15 |
|  | B1K-32-02 | wild | winter | Israel | 1 |
|  | B1K-32-17 | wild | n.d. | Israel | 4 |
|  | B1K-33-19 | wild | n.d. | Israel | 1 |
|  | B1K-35-04 | wild | n.d. | Israel | 1 |
|  | B1K-35-16 | wild | n.d. | Israel | 7 |
|  | B1K-37-19 | wild | n.d. | Israel | 5 |
|  | B1K-38-12 | wild | n.d. | Israel | 11 |
|  | B1K-38-14 | wild | n.d. | Israel | 5 |
|  | B1K-39-02 | wild | winter | Israel | 9 |
|  | B1K-39-20 | wild | n.d. | Israel | 9 |
|  | B1K-41-08 | wild | n.d. | Israel | 8 |
|  | B1K-41-18 | wild | n.d. | Israel | 8 |
|  | B1K-42-07 | wild | n.d. | Israel | 8 |
|  | B1K-42-17 | wild | n.d. | Israel | 1 |
|  | B1K-43-01 | wild | n.d. | Israel | 4 |
|  | B1K-43-14 | wild | n.d. | Israel | 14 |
|  | B1K-44-05 | wild | n.d. | Israel | 1 |
|  | B1K-45-08 | wild | winter | Israel | 8 |
|  | B1K-45-09 | wild | n.d. | Israel | 1 |
|  | B1K-46-07 | wild | n.d. | Israel | 4 |
|  | B1K-46-15 | wild | winter | Israel | 10 |
|  | B1K-47-13 | wild | n.d. | Israel | 12 |
|  | B1K-48-16 | wild | n.d. | Israel | 11 |
|  | B1K-48-19 | wild | n.d. | Israel | 12 |
|  | B1K-49-13 | wild | n.d. | Israel | 1 |
|  | B1K-49-18 | wild | n.d. | Israel | 7 |
| *agriocrithon* | HID383-2 | wild | n.d. | China | 4 |
|  | FT392 | wild | spring | China | 1 |
|  | B1K-52-01 | wild | spring | Israel | 1 |

* - B1K - Barley 1k collection (Hübner *et al.*, 2009); FT – collection (Comadran *et al.*, 2012); other genotypes are from the collection of Max Planck Institute for Plant Breeding Research, Cologne. Spring and winter growth type was determined by absence/presence of the *Vrn-H2* locus (Karsai *et al.*, 2005).

**Table S3a** Barley (*Hordeum vulgare*) flowering-related genes selected for targeted enrichment: genes extracted from NCBI Genbank

| NCBI accession # | Gene annotation |
| --- | --- |
| AJ249145 | *Hordeum vulgare* mRNA for MADS-box protein 7 (m7 gene) |
| AJ249146 | *Hordeum vulgare* mRNA for MADS-box protein 8 (m8 gene) |
| AJ249147 | *Hordeum vulgare* mRNA for MADS-box protein 9 (m9 gene) |
| AF460219 | *Hordeum vulgare* subsp. *vulgare* nuclear transcription factor SLN1 gene, complete cds |
| AF486648 | *Hordeum vulgare* subsp. *vulgare* AGAMOUS-like protein 1 HvAG1 (AG1) mRNA, complete cds |
| AF486649 | *Hordeum vulgare* subsp. *vulgare* AGAMOUS-like protein 2 HvAG2 (AG2) mRNA, complete cds |
| AJ312330 | *Hordeum vulgare* partial dof166 gene for dof zinc finger protein, exon 1 |
| AY082958 | *Hordeum vulgare* CONSTANS-like protein CO5 (CO5) gene, complete cds |
| AY082960 | *Hordeum vulgare* CONSTANS-like protein CO6 (CO6) gene, partial cds |
| AY082965 | *Hordeum vulgare* CONSTANS-like protein CO9 (CO9) gene, partial cds |
| AY082963 | *Hordeum vulgare* clone HV_CEb0009E08f CONSTANS-like protein CO7 (CO7) mRNA, partial cds |
| AY082964 | *Hordeum vulgare* CONSTANS-like protein CO8 (CO8) gene, complete cds |
| AF490467 | *Hordeum vulgare* subsp. *vulgare* cultivar Igri CONSTANS-like protein (CO1) gene, complete cds |
| AF490469 | *Hordeum vulgare* subsp. *vulgare* cultivar Igri CONSTANS-like protein (CO2) gene, complete cds |
| AF490473 | *Hordeum vulgare* subsp. *vulgare* cultivar Igri CONSTANS-like protein (CO3) gene, complete cds |
| AF521302 | *Hordeum vulgare* AP2 domain protein (DRF2) mRNA, complete cds |
| AY223807 | *Hordeum vulgare* AP2 transcriptional activator (DRF1) gene, complete cds, alternatively spliced |
| AY541065 | *Hordeum vulgare* subsp. *vulgare* APETALA3-like protein mRNA, complete cds |
| AY485977 | *Hordeum vulgare* cultivar Dairokkaku ZCCT-Ha (VRN2) gene, partial cds |
| AY485978 | *Hordeum vulgare* cultivar Dairokkaku ZCCT-Hb (VRN2) gene, partial cds |
| AY551428 | *Hordeum vulgare* subsp. *vulgare* GA 20-oxidase 1 (GA20ox1) mRNA, complete cds |
| AY551429 | *Hordeum vulgare* subsp. *vulgare* GA 20-oxidase 3 (GA20ox3) mRNA, complete cds |
| AY551430 | *Hordeum vulgare* subsp. *vulgare* GA 3-oxidase 1 (GA3ox1) mRNA, complete cds |
| AY551431 | *Hordeum vulgare* subsp. *vulgare* GA 3-oxidase 2 (GA3ox2) mRNA, complete cds |
| AY551432 | *Hordeum vulgare* subsp. *vulgare* GA 2-oxidase 4 (GA2ox4) mRNA, complete cds |
| AY551433 | *Hordeum vulgare* subsp. *vulgare* GA 2-oxidase 5 (GA2ox5) mRNA, complete cds |
| AY551435 | *Hordeum vulgare* subsp. *vulgare* copalyl diphosphate synthase-like protein (CPSL1) mRNA, complete cds |
| AY687931 | *Hordeum vulgare* ZCCT-Hc gene, partial cds |
| AY740524 | *Hordeum vulgare* subsp. *vulgare* gigantea-like protein (GI) gene, complete cds |
| AY970701 | *Hordeum vulgare* subsp. *vulgare* cultivar Igri pseudo-response regulator PPD-H1 (Ppd-H1) gene, complete cds |
| DQ100327 | *Hordeum vulgare* subsp. *vulgare* FT-like protein (FT1) gene, complete cds |
| DQ201140 | *Hordeum vulgare* subsp. *vulgare* cultivar Morex phytochrome A (PhyA) gene, complete cds |
| DQ201143 | *Hordeum vulgare* subsp. *vulgare* cultivar Morex phytochrome B (PhyB) gene, partial cds |
| DQ201149 | *Hordeum vulgare* subsp. *vulgare* cultivar Dicktoo cryptochrome 1a (Cry1a) gene, complete cds |
| DQ201152 | *Hordeum vulgare* subsp. *vulgare* cultivar Dicktoo cryptochrome 1b (Cry1b) gene, complete cds |
| DQ201155 | *Hordeum vulgare* subsp. *vulgare* cultivar Dicktoo cryptochrome 2 (Cry2) gene, partial cds |
| DQ238106 | *Hordeum vulgare* subsp. *vulgare* cv. Morex phytochrome C (PhyC) gene, complete cds |
| DQ297407 | *Hordeum vulgare* subsp. *vulgare* FT-like protein (FT2) gene, complete cds |
| DQ411319 | *Hordeum vulgare* subsp. *vulgare* FT-like protein 3 (FT3) gene, complete cds |
| DQ411320 | *Hordeum vulgare* subsp. *vulgare* FT-like protein 4 (FT4) gene, complete cds |
| DQ539338 | *Hordeum vulgare* subsp. *vulgare* terminal flower 1-like protein (TFL1) gene, complete cds |
| AB252049 | *Hordeum vulgare* Hvck2a mRNA for casein kinase II alpha, complete cds |
| AB252050 | *Hordeum vulgare* Hvck2b mRNA for casein kinase II beta, complete cds |
| EF043040 | *Hordeum vulgare* subsp. *vulgare* MADS-box protein 10 mRNA, complete cds |
| EF012202 | *Hordeum vulgare* subsp. *vulgare* FT-like protein (FT5) gene, complete cds |
| AM849822 | *Hordeum vulgare* mRNA for GID1-like gibberellin receptor (gse1 gene) |
| EU916968 | *Hordeum vulgare* ELF4-like protein mRNA, complete cds |
| FJ188402 | *Hordeum vulgare* flowering time control protein (FCA) mRNA, complete cds |

**Table S3b** Barley (*Hordeum vulgare*) flowering-related genes selected for targeted enrichment: unigenes extracted based on homology with flowering-related genes from *Brachypodium*.

| Barley unigene id, HarvEST 35 | Brachypodium flowering-related gene* | Homology-based annotation from different species* | | | |
| --- | --- | --- | --- | --- | --- |
|  |  | Brachypodium | Wheat/Barley | Arabidopsis | Rice |
| 966 | Bradi3g03040 | BdPAF | - | PAF1 | OsPAF |
| 2848 | Bradi4g38000 | BdSUF4 | - | SUF4 | OsSUF4 |
| 3015 | Bradi2g01020 | BdMFT2 | - | - | OsMFT2 |
| 3069 | Bradi4g35250 | BdFPA | - | FPA | OsFPA |
| 3108 | Bradi3g04140 | - | - | VIN3 | - |
| 3234 | Bradi5g14550 | - | - | ELF9 | OsELF9 |
| 3255 | Bradi1g21980 | - | TaVRN1 | AP1 | OsMADS14 |
| 3843 | Bradi4g02690 | BdFLKa | - | FLK | OsFLKa |
| 3889 | Bradi2g55550 | - | - | AtbZIP67 | - |
| 4088 | Bradi3g04040 | BdZTLb | TaZTL | LKP2 | OsZTLb |
| 4140 | Bradi4g43850 | BdFDL36 | TaFDL3 | - | - |
| 4604 | Bradi4g05950 | - | TmVIL1 | VRN5 | - |
| 4713 | Bradi3g14520 | BdFIE1 | - | FIE1 | OsFIE1a |
| 4834 | Bradi1g60030 | - | HvHap3 | HAP3B | - |
| 4835 | Bradi1g21900 | - | - | HAP3A | - |
| 5425 | Bradi2g59190 | BdMADS51-like | TaAGL41 | - | OsMADS51 |
| 5700 | Bradi2g22940 | - | - | NF-YB3 (HAP3) | - |
| 7973 | Bradi1g57640 | BdPIE1 | - | PIE1 | OsPIE1 |
| 9907 | Bradi5g21700 | - | - | FLC | OsMADS31 |
| 10361 | Bradi1g03880 | - | ZmIDS1 | TOE1 | - |
| 12240 | Bradi2g05900 | BdIDD2 | SbID1 | - | OsIDD2 |
| 13893 | Bradi1g45810 | BdVRT2 | HvVRT-2 | AGL24 | OsMADS55 |
| 14247 | Bradi3g39280 | - | - | NF-YC3 | - |
| 14250 | Bradi1g67980 | - | - | HAP5C | - |
| 14379 | Bradi3g38640 | - | - | GRF5 | OsGF14a |
| 14382 | Bradi1g11290 | - | - | GRF4 | OsGF14b |
| 14383 | Bradi4g16640 | - | - | GRF1 | OsGF14c |
| 14384 | Bradi3g46960 | - | - | GRF7 | OsGF14d |
| 14385 | Bradi3g36480 | - | - | GRF3 | OsGF14f |
| 16001 | Bradi3g57670 | BdCOP1 | - | COP1 | OsCOP1 |
| 16193 | Bradi2g02710 | - | - | TEM2 | OsRAV9 |
| 16194 | Bradi2g47220 | - | - | RAV1 | OsRAV11 |
| 16721 | Bradi3g33600 | BdPEP | - | PEP | OsPEP |
| 16771 | Bradi2g47940 | BdFVE | - | FVE | OsFVE |
| 17263 | Bradi3g42910 | BdSPY | OsSpindly | SPY | OsSPY |
| 17276 | Bradi2g37800 | - | ZmRAP2.7 Vgt1 | SMZ | - |
| 17379 | Bradi1g64460 | BdSWN | - | SWN | OsSWN |
| 17836 | Bradi1g13930 | BdMSI1 | - | MSI1 | OsMSI1 |
| 18157 | Bradi3g12900 | - | - | HUA2 | - |
| 18163 | Bradi1g32200 | - | - | HAP5B | - |
| 18730 | Bradi1g29920 | - | TaFDL15 | - | - |
| 18920 | Bradi2g60820 | BdFY | - | FY | OsFY |
| 19267 | Bradi3g00730 | - | - | AGL14 | - |
| 19311 | Bradi1g14320 | BdFLKb | - | - | OsFLKb |
| 19636 | Bradi2g62070 | BdLUX | - | LUX | OsLUX |
| 19711 | Bradi3g48880 | BdTOC1 | HvTOC1 | TOC1 | OsTOC1 |
| 19784 | Bradi1g72150 | - | HvVRT2 | SVP | OsMADS22 |
| 19844 | Bradi2g15800 | - | - | NF-YB8 (HAP3) | - |
| 20039 | Bradi3g03110 | - | - | VRN2 | - |
| 20082 | Bradi2g14290 | BdELF3 | TaELF3 | ELF3 | OsELF3 |
| 20272 | Bradi2g15900 | - | - | SPA2 | - |
| 20964 | Bradi2g17610 | - | - | RAV1-like | OsRAV12 |
| 21509 | Bradi4g16630 | BdFKF1 | TaFKF1 | FKF1 | OsFKF1 |
| 21639 | Bradi3g45730 | - | - | EFS | - |
| 21947 | Bradi4g27750 | BdPFT1 | - | PFT1 | OsPFT1 |
| 22244 | Bradi4g32090 | - | - | ABI5 | - |
| 22327 | Bradi1g46060 | - | - | ABF1 | - |
| 22370 | Bradi3g57960 | - | - | ABF4 | - |
| 22453 | Bradi1g17410 | - | TaDOF16 | CDF3 | - |
| 22503 | Bradi2g24120 | - | TaFDL6 | - | - |
| 23079 | Bradi3g60350 | - | - | CHE | - |
| 25214 | Bradi2g48660 | BdSPA1 | - | SPA1 | OsSPA1 |
| 25392 | Bradi3g24710 | BdLHP1 | - | LHP1 | OsLHP1 |
| 26657 | Bradi1g48340 | BdCLF | - | CLF | OsCLF |
| 26694 | Bradi2g10130 | BdARP6 | - | ARP6 | OsARP6 |
| 27247 | Bradi4g30090 | - | - | AGL-like | - |
| 27467 | Bradi2g60020 | BdIDD9 | - | - | OsIDD9 |
| 27523 | Bradi5g18210 | BdFLD | - | FLD | OsFLD |
| 27904 | Bradi1g63840 | - | - | AREB3 | - |
| 28593 | Bradi2g59940 | BdLD | - | LD | OsLD |
| 28971 | Bradi1g75000 | BdELF6 | - | ELF6 | OsELF6 |
| 29532 | Bradi5g20340 | BdLFY | - | LFY | OsLFY |
| 29576 | Bradi2g19930 | - | TaDOF19 | - | - |
| 29665 | Bradi2g48060 | BdLFL1 | - | - | OsLFL1 |
| 31448 | Bradi3g26910 | BdID1 | ZmID1 | - | OsID1 |
| 35596 | Bradi1g33450 | - | TmVIL2 | VEL3 | - |
| 37023 | Bradi3g38200 | - | - | ABF2 | - |
| 38800 | Bradi1g15310 | - | - | FRI-like | - |
| 40002 | Bradi2g09720 | - | HvCDF | CDF1 | - |
| 41599 | Bradi1g01520 | BdFRI | - | FRI | OsFRI |
| 42595 | Bradi2g36240 | - | TmVIL3 | VEL1 | - |
| 44106 | Bradi2g53060 | - | - | FDP | - |
| 47052 | Bradi1g77020 | BdSOC1 | TaSOC1 | SOC1 | OsMADS5 |
| 48985 | Bradi1g15320 | - | - | FRI-like | - |
| 49368 | Bradi2g58130 | BdREF6 | - | REF6 | OsREF6 |

* - Gene nomenclature and annotation according to Higgins et al. 2010.

**Table S4** PCR primers specific for barley *ARR*-like gene (Bowman contig_1987437)

| Primer name and sequence (5’ to 3’)^*^ | | | |
| --- | --- | --- | --- |
| Forward | | Reverse | |
| arr48369_1f | ctagatcgaagccggacgg | arr48369_1r | ggctacggggaggatatag |
| arr48369_2f | cggatcctgctttcccgg | arr48369_2r | agggaggggatgaggatg |
| arr48369_3f | gcagtgaccacggtggac | arr48369_3r | tcaaccatggtggctaggg |
| arr48369_4f | aggatcagcaggtcagcac | arr48369_4r | gaaacacatttcctgtggtctg |
| arr48369_5f | gctgtctagtagtttggcac | arr48369_5r | ggacaagcaaaagatacggtc |
| arr48369_6f | tttctgtggccacttggtgc | arr48369_6r | ttcctctttctgctgccgc |
| arr48369_7f | tcggcagcagcagaaagca | arr48369_7r | tatctacaacttggtcttcttcac |

* PCR reactions (1x HF buffer, 0.2 µM dNTPs, 1 µM primers, 1 U Phusion Hi-Fi polymerase (Thermo Scientific), 100 ng DNA) were incubated in the PTC DNA Engine thermocycler (Biorad) at the following conditions: 98^o^C for 3 min; 35 cycles of 98^o^C for 30 s, 61^o^C for 30 s, 72^o^C for 1 min; 72^o^C for 5 min.

**Supporting data**

**Notes S1** Sequencing of barley *ARR*-like gene

To identify alternative candidate genes residing in the putative location of the *eam10* QTL, we extracted 149 *Brachypodium* genes downstream of the marker ABC166 (Bradi2g60920.1; 155.85 cM) using the GenomeZipper and performed a functional annotation using Gene Ontology analysis implemented in the Blast2GO suite (Götz *et al.*, 2008; Table S5). Bradi2g61000, a gene residing ~16 cM above *HvLUX1*, was identified as a homolog of *Arabidopsis* response regulator (*ARR*) genes implicated in the regulation of circadian rhythms (Hazen *et al.*, 2005). Therefore, if a barley homolog of Bradi2g61000 has a different allele in Bowman(*eam10*) than in Bowman, this gene might be an alternative candidate explaining the observed flowering phenotypes and modification of expression patterns of other circadian genes. To test this hypothesis, we extracted the genomic contig, Bowman contig_1987437, carrying a barley homolog of the Brachypodium *ARR*-like gene Bradi2g61000 using the IPK barley BLAST server (http://webblast.ipk-gatersleben.de). The barley *ARR*-like gene and its promoter region were amplified from Bowman(*eam10*) using a set of specific primers (Supporting information Table S4). The PCR fragments were gel purified and Sanger-sequenced. Sequence analysis of a full-length gene and 850 bp of a promoter region of barley *ARR*-like gene revealed that Bowman(*eam10*) and Bowman carry identical alleles.

**Supporting Methods**

**Methods S1** Preparation of TruSeq libraries.

1. Shear 1 µg of genomic DNA samples to the size of 200-300 bp following the procedure described by Meyer & Kircher (2010).
2. End-repair the DNA fragments using the protocol by Meyer & Kircher (2010) adjusted to the final volume of 63 µL with extra enzyme deactivation step 20 min at 75ºC at the end of incubation (T4 polynucleotide kinase and polymerase supplied by NEB).
3. Perform A-tailing of the repaired libraries using the following protocol:

Reagent Volume, µL Final concentration

in 70-µL reaction

|  |
| --- |

End-repaired library 63

Klenow exo(-) (5 U/µL), NEB 2.8 0.2 U/µL

dATP 1 mM 0.56 8 µM

Water 3.46

Incubate the reactions 30 min at 37^o^C.

1. Purify the mixtures with 1:1 sample to Agencourt AMPure beads ratio following manufacturer’s recommendations (elute in 20 µL of water). Measure sample concentration using Quant-iT™ PicoGreen assay (Invitrogen) according to the manufacturer’s protocol.
2. Prepare mixes of barcoded TruSeq adapters according to Meyer & Kircher (2010) protocol. Ligate adapters to the libraries using the following protocol:

Reagent Volume, µL Final concentration

in 25-µL reaction

|  |
| --- |

T4 ligase buffer 10x 2.5 1x

Adapter 40 µM 1 1.6 µM

A-tailed library X (200 ng) 8 ng/µL

Water up to 24 µL

*mix thoroughly then add*

T4 DNA ligase, 5 U/µl (NEB) 1 0.2 U/µL

Incubate the reactions at 16^o^C overnight.

1. Purify the mixtures with 1:1 sample to Agencourt AMPure beads ratio following manufacturer’s recommendations (elute in 20 µL of water).
2. Pool individual libraries in equal volumes. Separate library fragments by size using standard agarose gel electrophoresis, excise a gel slice containing the fragments in the range of 300-400 bp, and purify the fragments using QIAquick gel purification kit (QIAGEN) following manufacturer’s recommendations.
3. Measure sample concentration using Quant-iT™ PicoGreen assay (Invitrogen) according to manufacturer’s protocol.

**Methods S2** Read processing workflow using the Galaxy server (http://galaxy.wur.nl)

| *Step 1: FastQ Groomer* | | | |
| --- | --- | --- | --- |
| Input FastQ quality scores type: Sanger | | | |
| *Step 2: FastQ Quality Trimmer* | | | |
| FastQ File: Output dataset from step 1  Keep reads with zero length: False  Trim ends: 5' and 3'  Window size: 5  Step size: 1  Maximum number of bases to exclude from the window during aggregation: 0  Aggregate action for window: mean of scores  Quality score: 10.0 | | | |
| *Step 3: Filter FastQ* | | | |
| FastQ file: Output dataset from step 2  Minimum size: 30 | | | |
| *Step 4: Remove sequencing artifacts* | | | |
| Library to filter: Output dataset from step 3 | | | |
| *Step 5: Contaminant QC and filtering* | | | |
| FastQ file to map/filter: Output dataset from step 4  Select genome sets for alignment:  /share/bowtie/indexes/Homo_sapiens.GRCh37  /share/bowtie/indexes/Ecoli_U00096  /share/bowtie/indexes/PhiX_NC001422.fasta  All reads or a subset: All sequences | | | |
| *Step 6: Parse bowtie hits* | | | |
| Bowtie table: Output dataset from step 5  How to deal with paired-end: No paired-end data or do not automatically add the non-mapping sequence of a pair | | | |
| *Step 7: FastQ_filter* | | | |
| FastQ reads: Output dataset from step 4  List of sequence IDs to filter: Output dataset from step 6 | | | |
| *Step 8: FastQ Groomer* | | | |
| File to groom: Output dataset from step 7  Input FastQ quality scores type: Sanger | | | |
| *Step 9: Cutadapt* | | | |
| FastQ file to trim: Output dataset from step 8  5’ or 3; (anywhere) Adapters 1  Source: Enter custom 5’ or 3’ adapter sequence  GATCGGAAGAGCGTCGTGTAGGGAAAGAGTGTAGATCTCGGTGGTCGCCGTATCATT  5’ or 3; (anywhere) Adapters 2  Source: Enter custom 5’ or 3’ adapter sequence  GATCGGAAGAGCACACGTCTGAACTCCAGTCAC  Maximum error rate: 0.15  Match times: 1  Minimum overlap length: 5  Discard trimmed reads: False  Minimum length: 30  **Methods S3** Polymorphism filtering workflow | | | |
| Filter | Passing value | Effect | Tools |
| Allele depth | > 15% (homozygous) or > 30% (heterozygous) of the mean depth of coverage | Filtering out SNP called at the low coverage regions | ‘SelectVariants’ GATK 2.1.3 |
| Ratio: SNP allele count/allele depth | > 0.6 (heterozygous)  1 (homozygous) | Alleviating the effect of PCR chimera formation during amplification of barcoded pooled libraries. All ‘heterozygous’ polymorphisms that passed the filters were treated as homozygous. | ‘SelectVariants’ GATK 2.1.3 and Microsoft Excel 2010 |
| SNP allele Phred-scaled likelihood | > than reference allele likelihood |  |  |
| Convert polymorphisms called as heterozygous into homozygous state | --- |  | Custom bash script |

**Methods S4** Permanent web links to the detailed results of the synteny analysis in the vicinity of *LUX* genes in three Poaceae species

CoGe database was searched for the following genes: *LUX1* locus – Sb03g047330, LOC_Os01g74020, and Bradi2g62070; *LUX2* locus - Sb03g039610, LOC_Os01g62660, and Bradi2g5479.

1. Synteny of the ancestral *LUX1* locus in rice*,* sorghum*,* and Brachypodium

http://genomevolution.org/CoGe/GEvo.pl?prog=blastz;iw=1600;fh=20;padding=2;colorfeat=1;nt=1;cbc=0;spike_len=15;skip_feat_overlap=1;skip_hsp_overlap=1;bzW=8;bzK=6000;bzO=400;bzE=30;accn1=Sb03g047330;fid1=19426091;dsid1=34580;dsgid1=93;chr1=3;dr1up=46199;dr1down=12525;ref1=0;accn2=LOC_Os01g74020.1;fid2=304023875;dsid2=66093;dsgid2=16888;chr2=Chr1;dr2up=37137;dr2down=83205;ref2=1;accn3=bradi2g62070;fid3=35290068;dsid3=40124;dsgid3=1607;chr3=Bd2;dr3up=44114;dr3down=66201;ref3=0;num_seqs=3;hsp_overlap_limit=0;hsp_size_limit=0

1. Synteny of the duplicated *LUX2* locus in rice, sorghum, and Brachypodium and its comparison with the rice *LUX1* locus

http://genomevolution.org/CoGe/GEvo.pl?prog=blastz;iw=1600;fh=20;padding=10;colorfeat=1;nt=1;cbc=0;spike_len=15;skip_feat_overlap=1;skip_hsp_overlap=1;bzW=8;bzK=6000;bzO=400;bzE=30;accn1=LOC_Os01g74020;fid1=304023875;dsid1=66093;dsgid1=16888;chr1=Chr1;dr1up=45397;dr1down=9271;ref1=0;accn2=LOC_Os01g62660.1;fid2=304016931;dsid2=66093;dsgid2=16888;chr2=Chr1;dr2up=35513;dr2down=10000;rev2=1;ref2=1;accn3=Bradi2g54790;fid3=35479227;dsid3=40124;dsgid3=1607;chr3=Bd2;dr3up=8903;dr3down=5158;rev3=1;ref3=0;num_seqs=3;hsp_overlap_limit=0;hsp_size_limit=300

**Supporting References**

**Götz S, García-Gómez JM, Terol J, Williams TD, Nagaraj SH, Nueda MJ, Robles M, Talón M, Dopazo J, Conesa A. (2008).** High-throughput functional annotation and data mining with the Blast2GO suite. *Nucleic Acids Research* **36:** 3420-3435.

**Karsai I, Szücs P, Mészáros K, Filichkina T, Hayes PM, Skinner JS, Láng L, Bedo Z. (2005).** The *Vrn-H2* locus is a major determinant of flowering time in a facultative winter growth habit barley (*Hordeum vulgare* L.) mapping population. *Theoretical and Applied Genetics* **110:** 1458–1466.
